# Supplementary material for: lncRNAs UC.145 and PRKG1-AS1 Determine the Functional Output of DKK1 in Regulating the Wnt Signaling Pathway in Gastric Cancer
Source: Cancers (Basel). 2022 May 11;14(10):2369. doi: 10.3390/cancers14102369 (PMC9140071; doi:10.3390/cancers14102369)
Supplement: Supplementary file 1 [file cancers-1699923-WB.pdf › cancers-1699923-WB.pdf]

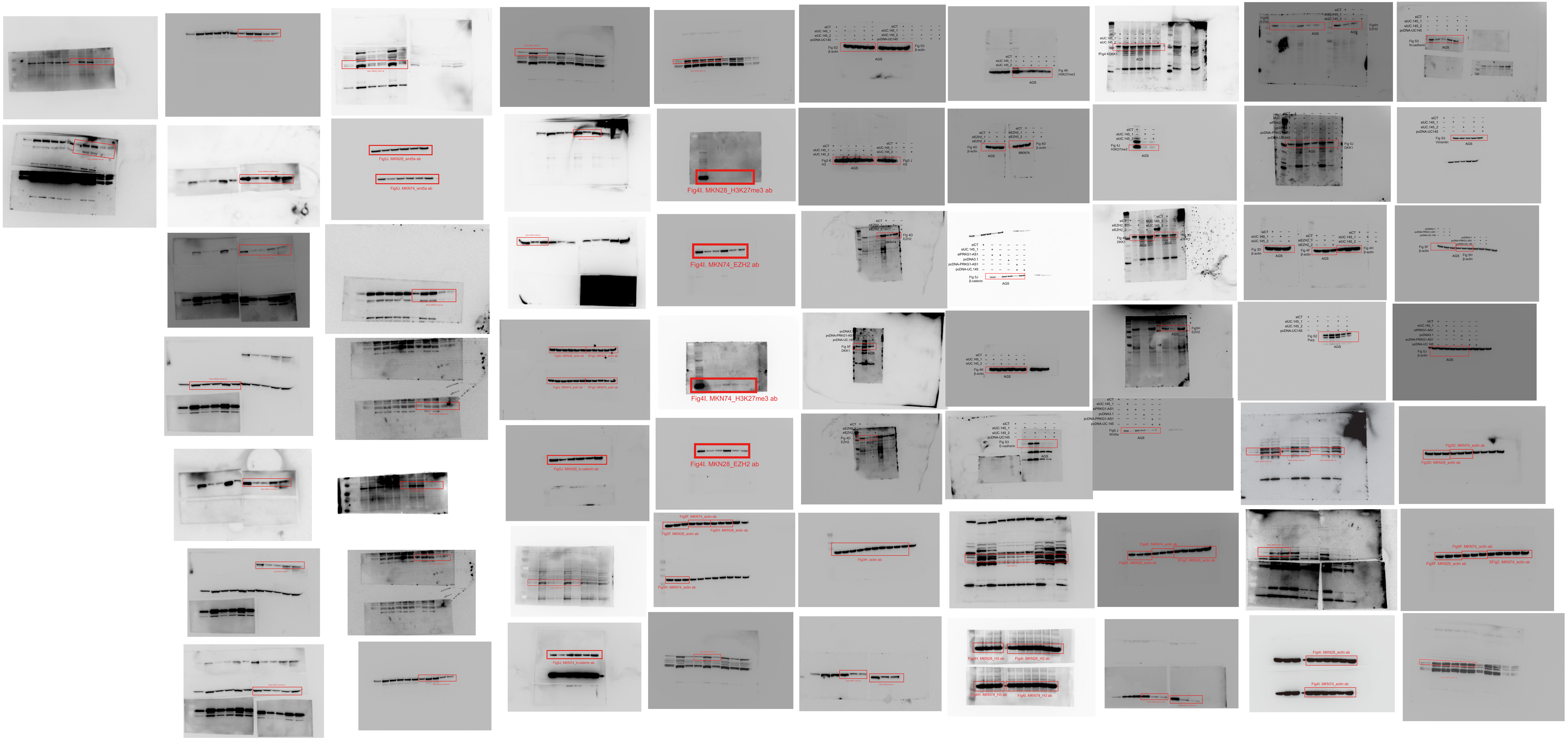

Fig5J. MKN28_wnt5a ab
Fig5J. MKN74_wnt5a ab
Fig4I. MKN28_H3K27me3 ab
Fig4I. MKN74_EZH2 ab
Fig4I. MKN74_H3K27me3 ab
Fig4I. MKN28_EZH2 ab
