# Supplementary material for: lncRNAs UC.145 and PRKG1-AS1 Determine the Functional Output of DKK1 in Regulating the Wnt Signaling Pathway in Gastric Cancer
Source: Cancers (Basel). 2022 May 11;14(10):2369. doi: 10.3390/cancers14102369 (PMC9140071; doi:10.3390/cancers14102369)
Supplement: Supplementary file 1 [file cancers-14-02369-s001.zip › cancers-1699923-supplementary.pdf]

Supplementary Information

Supplementary Figures and Legends

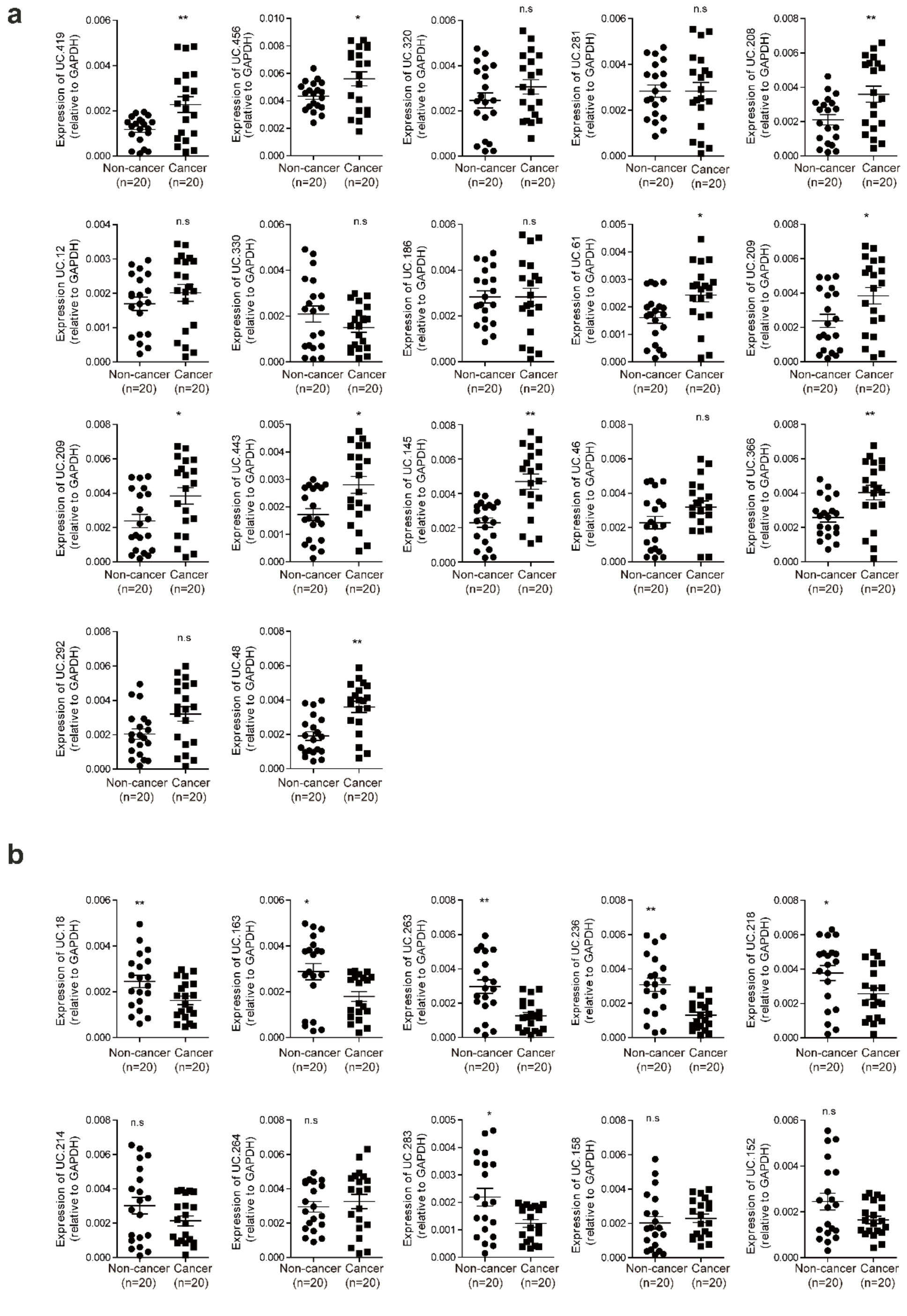

Figure S1. T-UCR validation. (**a**) Using microarray analysis results, 17 T-UCRs with increased mRNA expression were identified in 20 samples obtained from patients with gastric cancer (GC), measured using qRT-PCR. (**b**) Relative mRNA expression of ten T-UCRs with reduced expression in GC tissue measured using qRT-PCR. FC > 2.0, $p < 0.05$. Data are shown as mean ± SD of three independent experiments (* $p \leq 0.05$; ** $p \leq 0.01$).

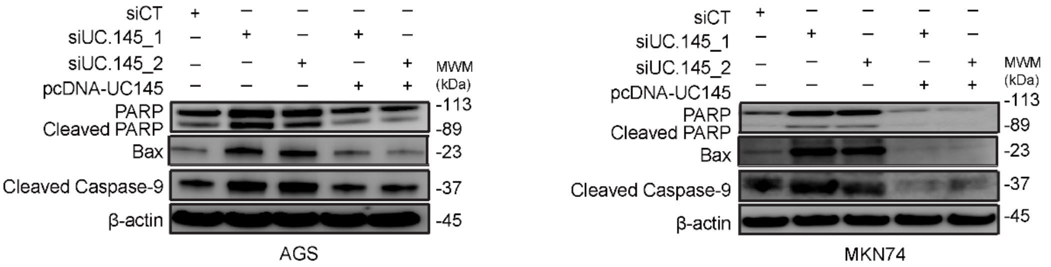

Figure S2. Effect of UC.145 on apoptosis. Western blot analysis of apoptosis marker protein levels in AGS (**left** panel) and MKN74 (**right** panel) cells transfected with siControl (siCT), siUC.145s, or pcDNA-UC.145.

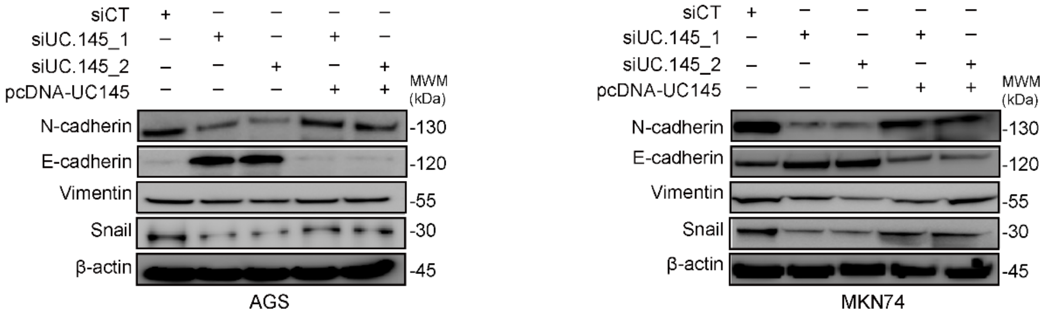

Figure S3. Effect of UC.145 on the EMT process of gastric cancer cells. Western blot analysis of EMT marker protein levels in AGS (**left** panel) and MKN74 (**right** panel) cells transfected with siControl (siCT), siUC.145s, or pcDNA-UC.145.

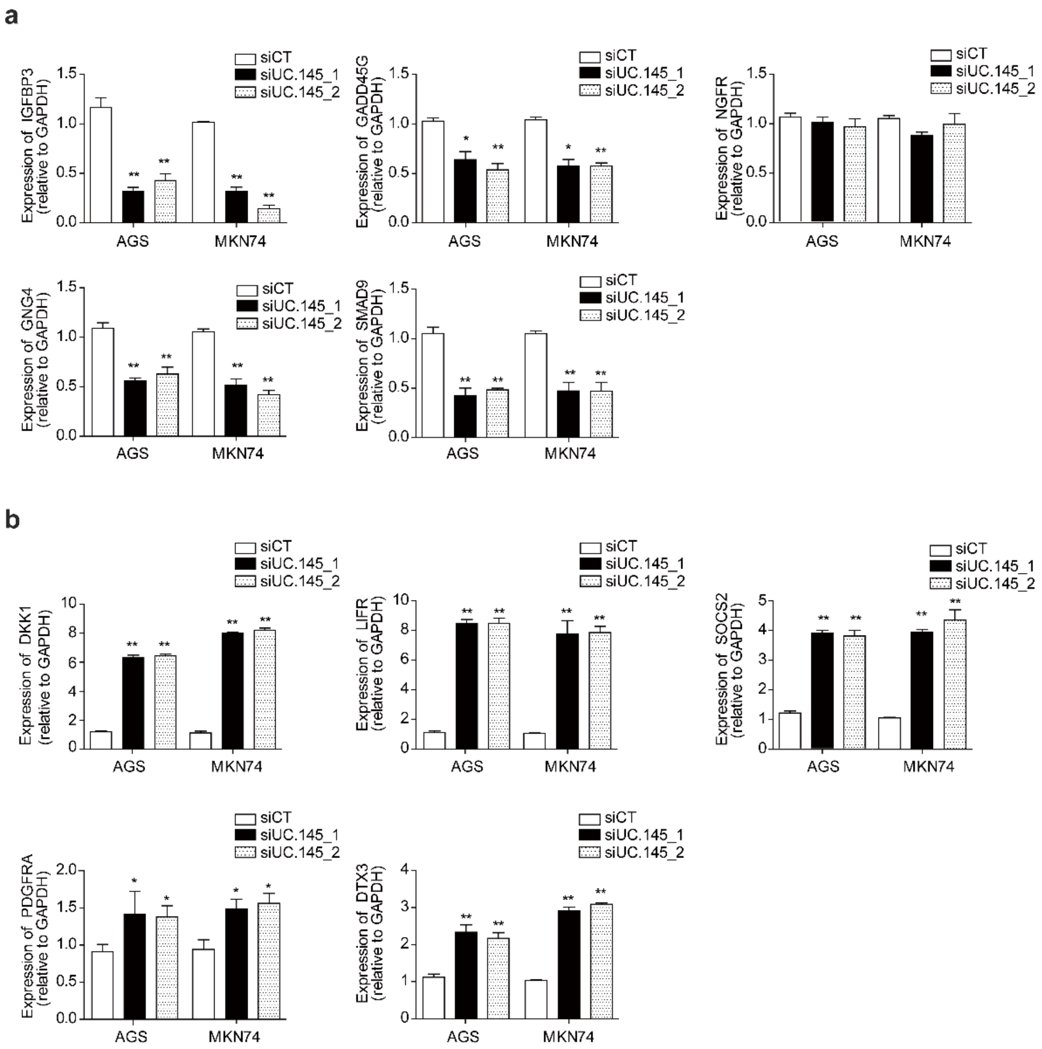

Figure S4. Differentially expressed genes in gastric cancer cells. Relative mRNA expression of (**a**) five downregulated genes and (**b**) five upregulated genes in AGS and MKN74 cells determined by Nanostring pathway analysis of putative target genes of UC.145, measured using qRT-PCR. Data are shown as mean ± SD of three independent experiments (* $p \leq 0.05$; ** $p \leq 0.01$).

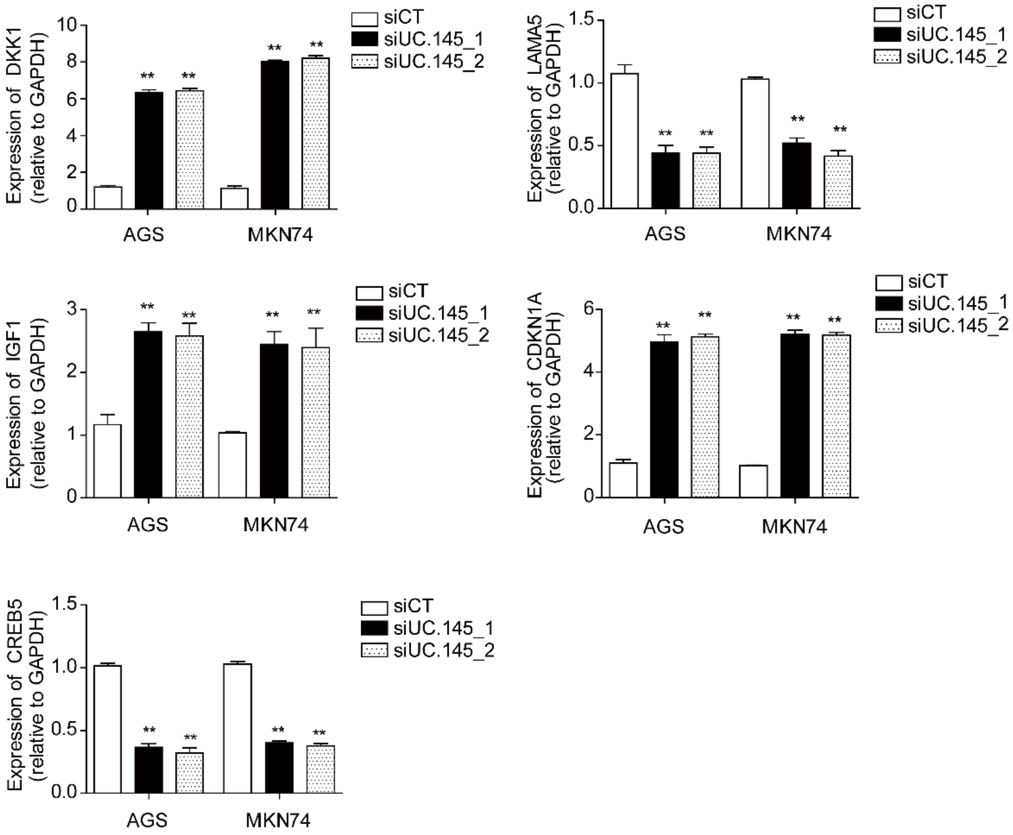

Figure S5. UC.145 target gene identification. Relative mRNA expression of five target genes of UC.145 in AGS and MKN74 cells based on Venn diagram-based analysis of a GEO dataset, measured using qRT-PCR. Data are shown as mean ± SD of three independent experiments (* $p \leq 0.05$; ** $p \leq 0.01$).

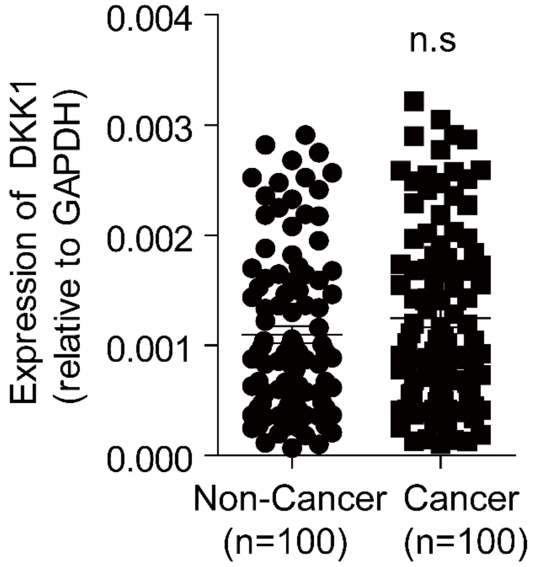

Figure S6. Confirmation of *DKK1* expression in gastric cancer (GC). Relative mRNA expression of *DKK1* in 100 pairs of tissue samples obtained from patients with GC, measured using qRT-PCR. n.s., not significant.

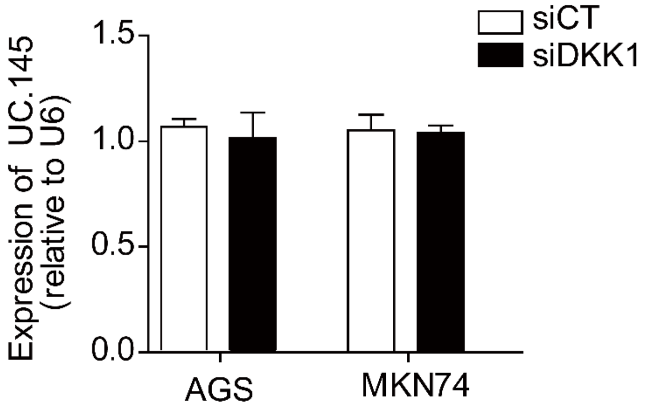

Figure S7. *DKK1* silencing affects UC.145 expression in gastric cancer cells. Relative mRNA expression of UC.145 in AGS and MKN74 cells transfected with siDKK1, measured using qRT-PCR.

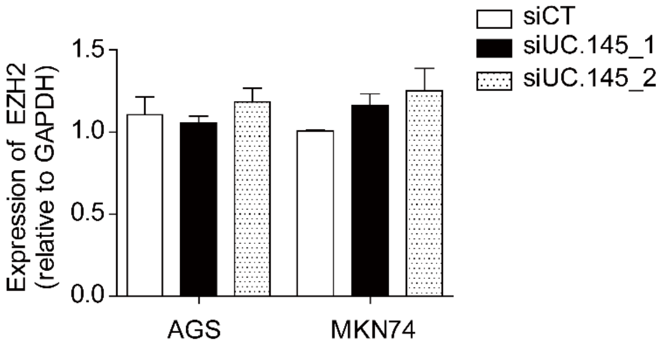

Figure S8. UC.145 affects EZH2 at the transcriptional level in GC cells. Relative mRNA expression of EZH2 in AGS and MKN74 cells, measured using qRT-PCR.

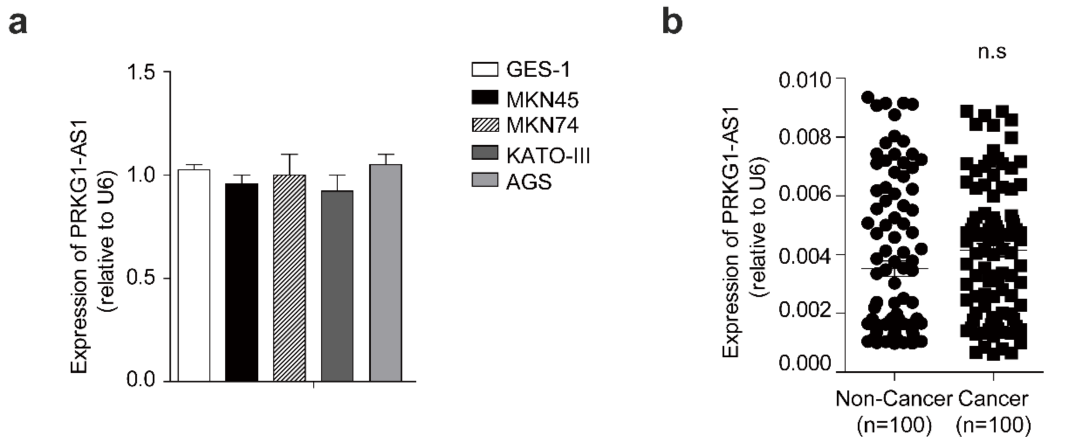

Figure S9. Confirmation of PRKG1-AS1 expression in gastric cancer (GC). (**a**) Relative mRNA expression of PRKG1-AS1 in the normal gastric cells (GES-1) compared with that in GC cells, measured using qRT-PCR; (**b**) Relative mRNA expression of PRKG-AS1 in 100 pairs of tissue samples obtained from patients with gastric cancer, measured using qRT-PCR. n.s., not significant.

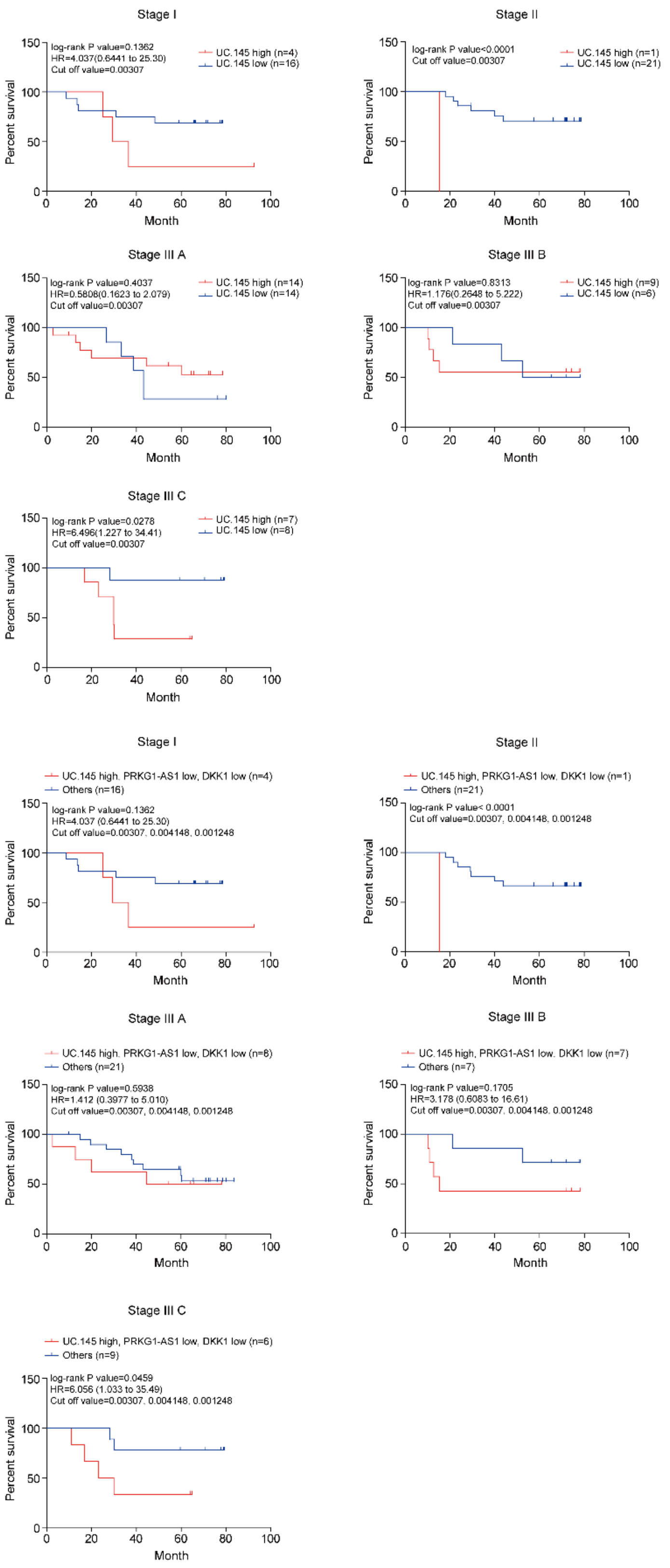

Figure S10. Survival stage analysis. Kaplan–Meier estimates of patient overall survival in each cancer stage using the number of cases in which each gene was either upregulated or downregulated.

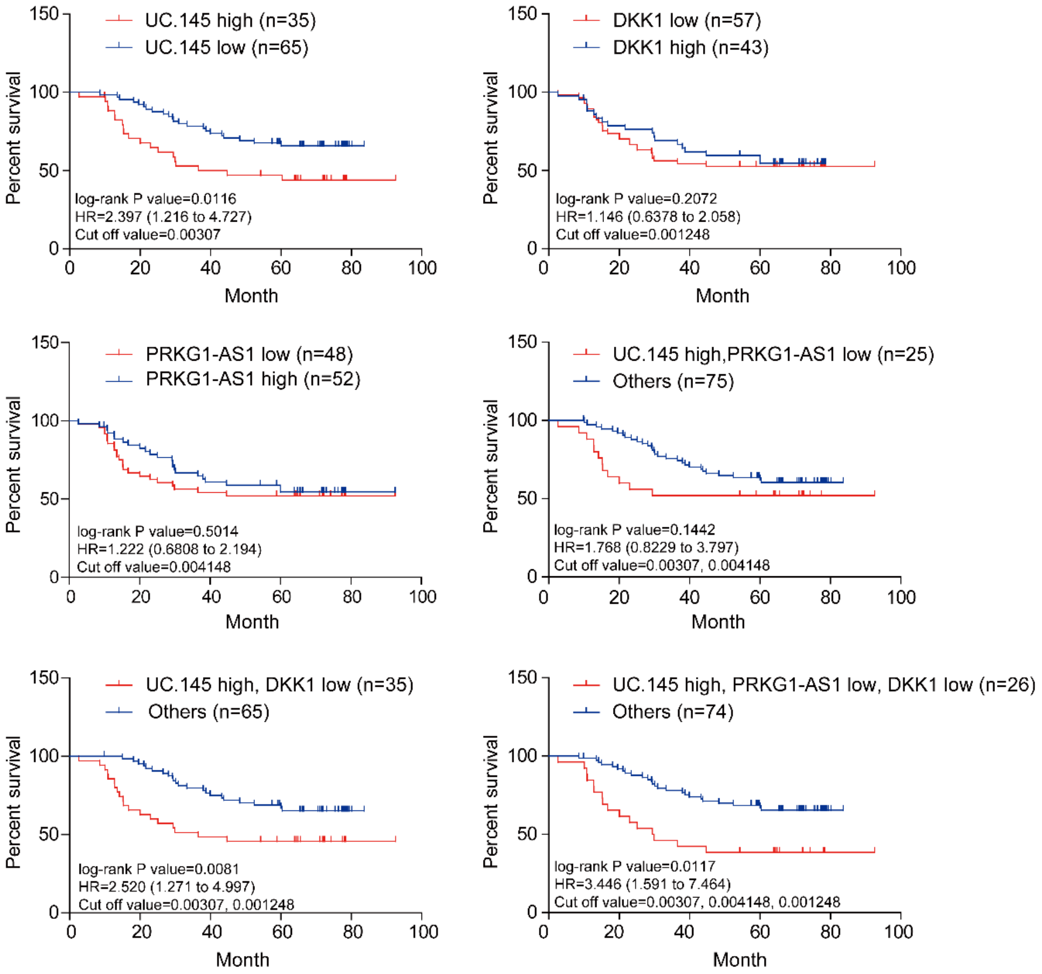

Figure S11. Survival analysis of UC.145, *DKK1*, and PRKG1-AS1. Kaplan–Meier estimates of patient overall survival using the number of cases in which each gene was either upregulated or downregulated.

Supplementary Tables

Table S1. Primer sequences used in this study.

Primer Name	Direction	Sequences (5' to 3')
	**Primer Sequences for qRT-PCR**	
UC.145	Forward	TTTCACTGCTGCTTGGTGTC
	Reverse	AGCCGGCACTAATAGTCCAA
UC.419	Forward	TCAAAGACACGAAGGGAATG
	Reverse	TTTTTAATTCAAACTGTTTTGTTCAGA
UC.456	Forward	CAGGGTGATGGTGAGGATTC
	Reverse	TGAGGTTTTTGGTGGTGGTT
UC.320	Forward	CAGGATATCACTGTGCAGAAATG
	Reverse	CCTTGGGTTGTCAAAAATGG
UC.281	Forward	CCGAATCCCTCTGTTGGTTA
	Reverse	TGAGGCCTTTATTGGAATTCTG
UC.208	Forward	TGGTTAGACAACACCCTCCAG
	Reverse	GGTGATCTGGTTCAAGTAGGG
UC.12	Forward	CAGTTCATTATAAGTAGTTGATGTCCA
	Reverse	GTGGATCGACTCATTGGTGA
UC.330	Forward	GTTCCGACCGGCGTTTAG
	Reverse	ACGGTAATCCAGCGAGGACT
UC.186	Forward	GGTGAATGGTATGCTTTCAACA
	Reverse	ATGCATCCTGTGTTCAGCAA
UC.61	Forward	CGCCCATGAAACAAAAACA
	Reverse	GGGTGTCTTCCTGTGCAACT
UC.209	Forward	TGATTTATCTTCCACATTCTTGGA
	Reverse	TTTTATAATCCCCACGTGCTT
UC.443	Forward	CTTGGCTTTTCTCCCTTGGT
	Reverse	ACCCTTGACTTTCCTTCAGC
UC.46	Forward	GGTCTTTACCTTATTGTACATTAGACC
	Reverse	CATGCCAAATTGCTTTTGCT
UC.366	Forward	AAAAATAAGGAGCCTTCTATTAAATCA
	Reverse	AGTGTACTGGGCCTTGGAGA
UC.48	Forward	ATGGGTGCTAGGCTGCTACT
	Reverse	TTTCTTCTTCAGTTGGCAGTTC
UC.18	Forward	GGAAAAGAGAAAAAGAGAAAGAAAAA
	Reverse	CATTTCCAGTGAAACCCACA
UC.163	Forward	GGGCAATTTTCTGTCTTCTTG
	Reverse	CATTTGGGATCCCCAGTTTC
UC.263	Forward	CACTGCTGTCTGGGACTGAA
	Reverse	TGCCAAACGGGTACCATACT
UC.236	Forward	GCCCTGCTTTGTTAGAGCAT
	Reverse	TTATCATCGCTGGGGAGAAC
UC.218	Forward	GCAAATTGCACTCTCTGCTCT
	Reverse	CTTCCCTTTCAAGCCCTCTA
UC.214	Forward	CAGAGACCCCTCTGCCTTTA
	Reverse	TCAATGTTCCTTGCCCTTTT
UC.264	Forward	CACTGCTGTCTGGGACTGAA
	Reverse	TGCCAAACGGGTACCATACT
UC.283	Forward	GGATCAAATCACATAAACTGCAA

	Reverse	CGCCAGGGAAGCACTTAATA
UC.158	Forward	ATCAGGCAGTTGGAAGCTCT
	Reverse	AGCCGCTCTCATTACAAACG
UC.152	Forward	AATGCCATAATGCCTCTCTGA
	Reverse	CTTTTAATGGCAGATTATCCCTGT
IGFBP3	Forward	AGTTGGGGGAATTAGGATGG
	Reverse	AATGGATTTGGCAGCTGTTC
GADD45G	Forward	TACGCTGATCCAGGCTTTCT
	Reverse	ACTCACCGAAATGAGGATGC
NGFR	Forward	AGCTCTGGGACAAGAGACCA
	Reverse	CCATGTTGACACCTGAAACG
GNG4	Forward	GCTTGAAAAACCTGCCTCAG
	Reverse	GGGTGCATGTGTGAAAACAG
SMAD9	Forward	GCTTGTTTGGGTCCAACTGT
	Reverse	AGCAGCAGTGGTAAGGGAGA
DKK1	Forward	TTGAGAAGGGGGATGATGAG
	Reverse	CCAGTCTGGCAAAACTGTGA
LIFR	Forward	CCAGTCCGTGGAGACATTTT
	Reverse	GGTGTGCCAATTAGGCTTGT
SOSC2	Forward	AAGGCTGAAGTCGCGTTTTA
	Reverse	AGTGAGGCCTGTGTCAGCTT
PDGFRA	Forward	TGGCTTCCTGGAGCTTAAAA
	Reverse	GAGTACCCTGGGGCACACTA
DTX3	Forward	TCTGTTTGAGCTGCATTTGG
	Reverse	TCCCAAGTCTGGCATTTAGG
LAMA5	Forward	CAGCCCCAAGCTGTCTACTC
	Reverse	GGCAGTGGTCACCTTTTGTT
IGF1	Forward	AGTCCCGCCAAAACACTATG
	Reverse	ATTTACAACCGCAGGGAGTG
CDKN1A	Forward	CTTCAAGGCAGTGGGAGAAG
	Reverse	TCTAAGCCCCACTGCTGTCT
CREB5	Forward	TTGAGCTGACGCTGAAGAGA
	Reverse	AGGGACACAGTTGGGTTCTG
EZH2	Forward	CAGTCACAGAGCCATGAGGA
	Reverse	CCCAGCATCTAGCAGTGTCA
DKK1 PROMOTER	Forward	TGCTTTTGCAGAGCCTATCA
	Reverse	GCAGGAACTCTGGGAACTTG
PRKG1-AS1	Forward	ACCTTTTACGGCCACATCAG
	Reverse	TGGCTGGTCCACATTGAATA
ACTB	Forward	GATGAGATTGGCATGGCTTT
	Reverse	CACCTTCACCGTTCCAGTTT
GAPDH	Forward	CCGGGAAACTGTGGCGTGATGG
	Reverse	AGGTGGAGGAGTGGGTGTCGCTGTT
U6	Forward	CTCGCTTCGGCAGCACA
	Reverse	AACGCTTCAGGAATTTGCGT
	**MS-PCR primers**	
*DKK1*_M	Forward	TTTGAATTCGGTTTTTAATTTTAAC
	Reverse	CAATAATCTAATACTTATTCCCGCC
*DKK1*_UM	Forward	TGAATTTGGTTTTTAATTTTAATGT
	Reverse	TCAATAATCTAATACTTATTCCCACC

Table S2. Nanostring nCounter expression results.

Gene Name	Accession No.	Probe Name	Class	%CV	Fold Change (siUC.145/siCT)
ABL1	NM_005157.3	NM_005157.3:3200	Endogenous	32.39	1.80
ACAD9	NM_014049.4	NM_014049.4:1935	Housekeeping	10.18	−1.19
ACVR1B	NM_004302.3	NM_004302.3:1700	Endogenous	25.93	−1.92
ACVR1C	NM_145259.2	NM_145259.2:5168	Endogenous	32.04	−1.71
ACVR2A	NM_001616.3	NM_001616.3:1245	Endogenous	43.35	2.19
AGK	NM_018238.3	NM_018238.3:816	Housekeeping	16.61	−1.01
AKT1	NM_005163.2	NM_005163.2:1772	Endogenous	50.07	2.12
AKT2	NM_001626.2	NM_001626.2:1450	Endogenous	20.65	1.28
AKT3	NM_181690.1	NM_181690.1:755	Endogenous	65.13	−6.04
ALK	NM_004304.3	NM_004304.3:3495	Endogenous	52.10	1.22
ALKBH2	NM_001001655.2	NM_001001655.2:907	Endogenous	13.10	−1.44
ALKBH3	NM_139178.3	NM_139178.3:690	Endogenous	27.22	1.83
AMER1	NM_152424.3	NM_152424.3:5755	Endogenous	24.37	1.62
AMH	NM_000479.3	NM_000479.3:1626	Endogenous	46.10	1.56
AMMECR1L	NM_001199140.1	NM_001199140.1:3564	Housekeeping	6.39	−1.06
ANGPT1	NM_001146.3	NM_001146.3:2080	Endogenous	51.31	−3.04
APC	NM_000038.3	NM_000038.3:6850	Endogenous	24.26	1.41
APH1B	NM_001145646.1	NM_001145646.1:2930	Endogenous	52.46	3.13
AR	NM_001011645.1	NM_001011645.1:810	Endogenous	53.28	−1.12
ARID1A	NM_006015.4	NM_006015.4:5495	Endogenous	7.85	1.18
ARID1B	NM_020732.3	NM_020732.3:6335	Endogenous	10.05	1.07
ARID2	NM_152641.2	NM_152641.2:3355	Endogenous	43.54	1.66
ARNT2	NM_014862.3	NM_014862.3:5260	Endogenous	21.01	1.17
ASXL1	NM_001164603.1	NM_001164603.1:472	Endogenous	2.24	−1.01
ATM	NM_138292.3	NM_138292.3:6688	Endogenous	33.13	−2.53
ATR	NM_001184.2	NM_001184.2:565	Endogenous	17.10	−1.41
ATRX	NM_000489.3	NM_000489.3:139	Endogenous	2.31	−1.15
AXIN1	NM_181050.1	NM_181050.1:135	Endogenous	11.28	−1.14
AXIN2	NM_004655.3	NM_004655.3:1035	Endogenous	116.54	5.74
B2M	NM_004048.2	NM_004048.2:25	Endogenous	41.88	−1.06
BAD	NM_004322.3	NM_004322.3:652	Endogenous	29.69	−1.85
BAIAP3	NM_003933.4	NM_003933.4:1950	Endogenous	60.37	−8.23
BAMBI	NM_012342.2	NM_012342.2:832	Endogenous	104.57	4.48
BAP1	NM_004656.2	NM_004656.2:240	Endogenous	46.24	2.12
BAX	NM_138761.3	NM_138761.3:342	Endogenous	72.17	2.76
BCL2	NM_000657.2	NM_000657.2:5	Endogenous	56.78	−2.30

BCL2A1	NM_004049.2	NM_004049.2:80	Endogenous	16.84	−1.28
BCL2L1	NM_138578.1	NM_138578.1:1560	Endogenous	6.66	1.29
BCOR	NM_001123383.1	NM_001123383.1:1630	Endogenous	21.52	1.34
BDNF	NM_170732.4	NM_170732.4:730	Endogenous	30.11	−2.74
BID	NM_197966.1	NM_197966.1:2095	Endogenous	30.81	−1.95
BIRC3	NM_182962.1	NM_182962.1:3	Endogenous	46.06	1.56
BIRC7	NM_022161.2	NM_022161.2:1168	Endogenous	47.72	2.34
BMP2	NM_001200.2	NM_001200.2:1515	Endogenous	109.44	10.42
BMP4	NM_001202.2	NM_001202.2:490	Endogenous	90.74	3.25
BMP5	NM_021073.2	NM_021073.2:1570	Endogenous	42.63	1.25
BMP6	NM_001718.2	NM_001718.2:1045	Endogenous	89.41	−1.28
BMP7	NM_001719.1	NM_001719.1:525	Endogenous	59.25	−4.40
BMP8A	NM_181809.3	NM_181809.3:5015	Endogenous	22.53	3.13
BMPR1B	NM_001203.1	NM_001203.1:430	Endogenous	23.98	1.34
BNIP3	NM_004052.2	NM_004052.2:325	Endogenous	19.85	2.34
BRAF	NM_004333.3	NM_004333.3:565	Endogenous	18.67	−1.12
BRCA1	NM_007305.2	NM_007305.2:1275	Endogenous	7.86	−1.32
BRCA2	NM_000059.3	NM_000059.3:115	Endogenous	26.62	1.30
BRIP1	NM_032043.1	NM_032043.1:1130	Endogenous	13.61	−1.10
C10orf76	NM_024541.2	NM_024541.2:3750	Housekeeping	3.43	1.20
C19orf40	NM_152266.3	NM_152266.3:376	Endogenous	5.15	−1.17
CACNA1C	NM_199460.2	NM_199460.2:4785	Endogenous	56.90	2.08
CACNA1D	NM_000720.2	NM_000720.2:5195	Endogenous	100.52	4.17
CACNA1E	NM_000721.2	NM_000721.2:9325	Endogenous	61.03	1.25
CACNA1G	NM_198397.1	NM_198397.1:1380	Endogenous	67.22	−6.40
CACNA1H	NM_021098.2	NM_021098.2:3329	Endogenous	45.07	−2.24
CACNA2D1	NM_000722.2	NM_000722.2:335	Endogenous	48.27	−1.54
CACNA2D2	NM_001005505.1	NM_001005505.1:2045	Endogenous	61.74	−1.28
CACNA2D3	NM_018398.2	NM_018398.2:205	Endogenous	29.99	−1.92
CACNA2D4	NM_001005737.1	NM_001005737.1:990	Endogenous	56.53	−1.28
CACNB2	NM_000724.3	NM_000724.3:2610	Endogenous	28.18	−1.49
CACNB3	NM_000725.2	NM_000725.2:1115	Endogenous	27.03	−1.08
CACNB4	NM_001005747.2	NM_001005747.2:1483	Endogenous	29.99	−2.24
CACNG1	NM_000727.2	NM_000727.2:464	Endogenous	61.03	3.13
CACNG4	NM_014405.2	NM_014405.2:1030	Endogenous	20.25	−1.12
CACNG6	NM_145814.1	NM_145814.1:1745	Endogenous	55.44	−4.18
CALML3	NM_005185.2	NM_005185.2:685	Endogenous	57.73	−5.12
CALML5	NM_017422.4	NM_017422.4:136	Endogenous	11.51	1.56
CALML6	NM_138705.2	NM_138705.2:682	Endogenous	44.09	2.34
CAMK2B	NM_001220.3	NM_001220.3:365	Endogenous	71.62	−3.84

CAPN2	NM_001748.4	NM_001748.4:2085	Endogenous	29.01	1.68
CARD11	NM_032415.2	NM_032415.2:1075	Endogenous	15.87	−1.22
CASP10	NM_032977.3	NM_032977.3:20	Endogenous	10.12	1.56
CASP12	NM_001191016.1	NM_001191016.1:595	Endogenous	18.17	1.56
CASP3	NM_032991.2	NM_032991.2:685	Endogenous	4.79	1.09
CASP7	NM_001227.3	NM_001227.3:915	Endogenous	16.12	1.11
CASP8	NM_001228.4	NM_001228.4:301	Endogenous	11.83	−1.10
CASP9	NM_001229.2	NM_001229.2:1805	Endogenous	31.92	−1.05
CBL	NM_005188.2	NM_005188.2:7485	Endogenous	20.99	−1.12
CBLC	NM_012116.3	NM_012116.3:652	Endogenous	19.11	−1.18
CC2D1B	NM_032449.2	NM_032449.2:4182	Housekeeping	18.85	1.12
CCNA1	NM_003914.3	NM_003914.3:1605	Endogenous	47.66	1.17
CCNA2	NM_001237.2	NM_001237.2:1210	Endogenous	15.06	−1.42
CCNB1	NM_031966.2	NM_031966.2:715	Endogenous	8.75	−1.34
CCNB3	NM_033671.1	NM_033671.1:35	Endogenous	22.18	−1.28
CCND1	NM_053056.2	NM_053056.2:690	Endogenous	17.55	1.16
CCND2	NM_001759.2	NM_001759.2:5825	Endogenous	70.20	−5.63
CCND3	NM_001760.2	NM_001760.2:1215	Endogenous	24.60	−1.62
CCNE1	NM_001238.1	NM_001238.1:1635	Endogenous	76.76	2.95
CCNE2	NM_057735.1	NM_057735.1:50	Endogenous	42.95	2.61
CCNO	NM_021147.3	NM_021147.3:45	Endogenous	10.12	−1.28
CCR7	NM_001838.2	NM_001838.2:1610	Endogenous	15.87	−1.28
CD14	NM_000591.2	NM_000591.2:885	Endogenous	54.97	−1.28
CD19	NM_001770.4	NM_001770.4:1770	Endogenous	50.96	1.56
CD40	NM_001250.4	NM_001250.4:1265	Endogenous	115.37	2.34
CDC14A	NM_033313.2	NM_033313.2:1400	Endogenous	13.58	−1.16
CDC14B	NM_003671.3	NM_003671.3:4940	Endogenous	69.15	2.67
CDC25A	NM_001789.2	NM_001789.2:690	Endogenous	68.12	2.01
CDC25B	NM_021873.2	NM_021873.2:3045	Endogenous	5.32	−1.27
CDC25C	NM_001790.2	NM_001790.2:1055	Endogenous	45.84	−2.46
CDC6	NM_001254.3	NM_001254.3:1300	Endogenous	13.42	1.15
CDC7	NM_003503.2	NM_003503.2:805	Endogenous	18.70	−1.85
CDH1	NM_004360.2	NM_004360.2:1230	Endogenous	59.18	−3.72
CDK2	NM_001798.2	NM_001798.2:220	Endogenous	33.23	−2.30
CDK4	NM_000075.2	NM_000075.2:1055	Endogenous	5.12	−1.05
CDK6	NM_001259.5	NM_001259.5:15	Endogenous	62.10	1.63
CDKN1A	NM_000389.2	NM_000389.2:1975	Endogenous	68.32	2.68
CDKN1B	NM_004064.2	NM_004064.2:365	Endogenous	12.30	1.10
CDKN1C	NM_000076.2	NM_000076.2:1605	Endogenous	43.87	−2.88
CDKN2A	NM_000077.3	NM_000077.3:975	Endogenous	46.11	−2.56

CDKN2B	NM_004936.3	NM_004936.3:1175	Endogenous	89.06	5.59
CDKN2C	NM_001262.2	NM_001262.2:1295	Endogenous	11.28	−1.44
CDKN2D	NM_001800.3	NM_001800.3:870	Endogenous	41.77	−2.95
CEBPA	NM_004364.2	NM_004364.2:1320	Endogenous	72.78	2.41
CEBPE	NM_001805.2	NM_001805.2:1096	Endogenous	74.07	−2.56
CHAD	NM_001267.2	NM_001267.2:810	Endogenous	25.94	−1.92
CHEK1	NM_001114121.1	NM_001114121.1:2225	Endogenous	20.36	−1.99
CHEK2	NM_007194.3	NM_007194.3:140	Endogenous	33.78	−2.11
CHUK	NM_001278.3	NM_001278.3:860	Endogenous	12.11	1.26
CIC	NM_015125.3	NM_015125.3:1290	Endogenous	29.84	1.36
CLCF1	NM_013246.2	NM_013246.2:1296	Endogenous	28.25	2.34
CNOT10	NM_001256741.1	NM_001256741.1:1962	Housekeeping	12.26	1.00
CNOT4	NM_001190848.1	NM_001190848.1:795	Housekeeping	7.21	1.16
CNTFR	NM_147164.1	NM_147164.1:784	Endogenous	28.79	−1.65
COG7	NM_153603.3	NM_153603.3:1492	Housekeeping	37.92	−2.08
COL11A1	NM_001854.3	NM_001854.3:674	Endogenous	19.31	−1.28
COL11A2	NM_001163771.1	NM_001163771.1:760	Endogenous	52.19	−6.40
COL1A1	NM_000088.3	NM_000088.3:5210	Endogenous	34.04	−2.21
COL1A2	NM_000089.3	NM_000089.3:2635	Endogenous	57.58	1.17
COL24A1	NM_152890.5	NM_152890.5:4752	Endogenous	47.22	−1.92
COL27A1	NM_032888.2	NM_032888.2:5120	Endogenous	131.64	6.54
COL2A1	NM_001844.4	NM_001844.4:4745	Endogenous	17.34	−1.04
COL3A1	NM_000090.3	NM_000090.3:180	Endogenous	30.11	2.34
COL4A3	NM_000091.3	NM_000091.3:5630	Endogenous	39.31	−1.92
COL4A4	NM_000092.4	NM_000092.4:8255	Endogenous	12.31	1.95
COL4A5	NM_033381.1	NM_033381.1:5360	Endogenous	145.81	8.54
COL4A6	NM_001847.2	NM_001847.2:1070	Endogenous	14.80	1.35
COL5A1	NM_000093.3	NM_000093.3:6345	Endogenous	31.94	−1.02
COL5A2	NM_000393.3	NM_000393.3:4075	Endogenous	32.95	−1.92
COL6A6	NM_001102608.1	NM_001102608.1:5926	Endogenous	15.87	−1.28
COMP	NM_000095.2	NM_000095.2:1744	Endogenous	42.13	2.34
CREB3L1	NM_052854.1	NM_052854.1:195	Endogenous	63.35	2.27
CREB3L3	NM_001271995.1	NM_001271995.1:1050	Endogenous	82.23	1.37
CREB3L4	NM_130898.2	NM_130898.2:1505	Endogenous	36.40	−1.37
CREB5	NM_182898.2	NM_182898.2:1885	Endogenous	65.35	3.56
CREBBP	NM_004380.2	NM_004380.2:8855	Endogenous	57.13	1.30
CRLF2	NM_001012288.1	NM_001012288.1:605	Endogenous	15.91	−1.60
CSF1R	NM_005211.2	NM_005211.2:3775	Endogenous	16.56	−1.28
CSF2	NM_000758.2	NM_000758.2:475	Endogenous	64.06	2.34
CSF3	NM_000759.3	NM_000759.3:851	Endogenous	35.31	−1.28

CSF3R	NM_156038.2	NM_156038.2:90	Endogenous	64.94	1.56
CTNNB1	NM_001904.3	NM_001904.3:2265	Endogenous	6.22	−1.10
CUL1	NM_003592.2	NM_003592.2:1487	Endogenous	10.39	−1.20
CXXC4	NM_025212.1	NM_025212.1:293	Endogenous	56.34	1.17
CYLD	NM_015247.1	NM_015247.1:2890	Endogenous	46.64	−2.88
DAXX	NM_001350.3	NM_001350.3:1875	Endogenous	1.40	−1.14
DDB2	NM_000107.1	NM_000107.1:840	Endogenous	89.74	3.11
DDIT3	NM_004083.4	NM_004083.4:40	Endogenous	22.71	−1.41
DDIT4	NM_019058.2	NM_019058.2:85	Endogenous	67.66	−5.87
DDX50	NM_024045.1	NM_024045.1:1185	Housekeeping	5.77	−1.26
DHX16	NM_001164239.1	NM_001164239.1:2490	Housekeeping	8.09	−1.26
DKK1	NM_012242.2	NM_012242.2:75	Endogenous	166.87	49.67
DKK2	NM_014421.2	NM_014421.2:1135	Endogenous	38.46	−1.28
DKK4	NM_014420.2	NM_014420.2:640	Endogenous	41.87	1.30
DLL1	NM_005618.3	NM_005618.3:2580	Endogenous	45.87	−1.28
DLL3	NM_203486.2	NM_203486.2:1842	Endogenous	72.50	−7.25
DLL4	NM_019074.2	NM_019074.2:893	Endogenous	53.11	2.34
DNAJC14	NM_032364.5	NM_032364.5:1166	Housekeeping	17.03	−1.31
DNMT1	NM_001379.2	NM_001379.2:1495	Endogenous	12.97	−1.26
DNMT3A	NM_022552.3	NM_022552.3:3835	Endogenous	29.65	1.52
DTX1	NM_004416.2	NM_004416.2:2855	Endogenous	34.79	1.17
DTX3	NM_178502.2	NM_178502.2:1620	Endogenous	124.18	14.06
DTX4	NM_015177.1	NM_015177.1:4820	Endogenous	17.76	1.07
DUSP10	NM_144728.2	NM_144728.2:1176	Endogenous	10.35	1.49
DUSP2	NM_004418.3	NM_004418.3:1235	Endogenous	123.67	7.97
DUSP4	NM_057158.2	NM_057158.2:3115	Endogenous	113.52	9.38
DUSP5	NM_004419.3	NM_004419.3:675	Endogenous	40.64	1.56
DUSP6	NM_001946.2	NM_001946.2:1535	Endogenous	12.23	−1.68
DUSP8	NM_004420.2	NM_004420.2:2165	Endogenous	63.41	4.93
E2F1	NM_005225.1	NM_005225.1:935	Endogenous	19.61	−1.91
E2F5	NM_001951.3	NM_001951.3:444	Endogenous	53.58	2.38
EDC3	NM_001142443.1	NM_001142443.1:925	Housekeeping	17.11	−1.23
EFNA1	NM_004428.2	NM_004428.2:650	Endogenous	62.37	2.99
EFNA2	NM_001405.3	NM_001405.3:1538	Endogenous	92.05	2.59
EFNA3	NM_004952.4	NM_004952.4:1672	Endogenous	23.81	1.21
EFNA5	NM_001962.2	NM_001962.2:5035	Endogenous	20.37	−1.60
EGF	NM_001963.3	NM_001963.3:3930	Endogenous	67.69	−3.20
EGFR	NM_201282.1	NM_201282.1:360	Endogenous	40.36	−2.39
EIF2B4	NM_172195.3	NM_172195.3:1390	Housekeeping	7.37	1.24
EIF4EBP1	NM_004095.3	NM_004095.3:363	Endogenous	27.52	−1.93

ENDOG	NM_004435.2	NM_004435.2:694	Endogenous	4.66	1.06
EP300	NM_001429.2	NM_001429.2:715	Endogenous	32.80	−1.79
EPHA2	NM_004431.2	NM_004431.2:1525	Endogenous	32.13	1.80
EPO	NM_000799.2	NM_000799.2:1055	Endogenous	20.49	1.17
EPOR	NM_000121.2	NM_000121.2:1295	Endogenous	16.54	−1.06
ERBB2	NM_004448.2	NM_004448.2:2380	Endogenous	11.50	1.11
ERCC2	NM_000400.2	NM_000400.2:240	Endogenous	35.62	1.74
ERCC3	NM_000122.1	NM_000122.1:1950	Housekeeping	16.48	1.09
ERCC6	NM_000124.2	NM_000124.2:3235	Endogenous	37.82	2.02
ETS2	NM_005239.4	NM_005239.4:1175	Endogenous	28.97	1.45
ETV1	NM_004956.4	NM_004956.4:1719	Endogenous	22.00	−1.60
ETV4	NM_001079675.1	NM_001079675.1:1535	Endogenous	46.30	−2.94
ETV7	NM_016135.2	NM_016135.2:794	Endogenous	20.64	−2.13
EYA1	NM_172059.2	NM_172059.2:1090	Endogenous	24.20	−1.28
EZH2	NM_004456.3	NM_004456.3:190	Endogenous	14.83	−1.58
FANCA	NM_000135.2	NM_000135.2:265	Endogenous	64.69	3.02
FANCB	NM_152633.2	NM_152633.2:2470	Endogenous	11.90	1.10
FANCC	NM_000136.2	NM_000136.2:310	Endogenous	8.64	−1.19
FANCE	NM_021922.2	NM_021922.2:1275	Endogenous	29.76	−1.78
FANCF	NM_022725.2	NM_022725.2:845	Endogenous	6.53	−1.01
FANCG	NM_004629.1	NM_004629.1:1900	Endogenous	37.36	−2.38
FANCL	NM_001114636.1	NM_001114636.1:446	Endogenous	26.08	−1.67
FAS	NM_152876.1	NM_152876.1:1740	Endogenous	98.98	3.05
FASLG	NM_000639.1	NM_000639.1:625	Endogenous	24.53	1.30
FBXW7	NM_018315.4	NM_018315.4:1480	Endogenous	18.58	−1.06
FCF1	NM_015962.4	NM_015962.4:1022	Housekeeping	11.58	1.96
FEN1	NM_004111.4	NM_004111.4:425	Endogenous	12.75	−1.38
FGF1	NM_033137.1	NM_033137.1:315	Endogenous	80.08	1.76
FGF10	NM_004465.1	NM_004465.1:85	Endogenous	19.71	1.04
FGF11	NM_004112.2	NM_004112.2:1670	Endogenous	10.40	−1.00
FGF12	NM_004113.4	NM_004113.4:685	Endogenous	53.11	−3.61
FGF13	NM_033642.1	NM_033642.1:620	Endogenous	19.71	3.13
FGF14	NM_004115.3	NM_004115.3:725	Endogenous	10.12	−1.92
FGF16	NM_003868.1	NM_003868.1:504	Endogenous	41.43	−2.24
FGF17	NM_003867.2	NM_003867.2:279	Endogenous	20.25	−1.92
FGF18	NM_003862.1	NM_003862.1:850	Endogenous	65.31	2.26
FGF19	NM_005117.2	NM_005117.2:786	Endogenous	20.13	−1.08
FGF2	NM_002006.4	NM_002006.4:620	Endogenous	61.74	−2.56
FGF20	NM_019851.1	NM_019851.1:390	Endogenous	27.42	1.82
FGF21	NM_019113.2	NM_019113.2:125	Endogenous	14.44	1.95

FGF22	NM_020637.1	NM_020637.1:206	Endogenous	53.15	1.56
FGF23	NM_020638.2	NM_020638.2:1390	Endogenous	98.10	−1.28
FGF3	NM_005247.2	NM_005247.2:692	Endogenous	47.17	−1.49
FGF4	NM_002007.2	NM_002007.2:688	Endogenous	18.89	1.30
FGF5	NM_004464.3	NM_004464.3:4785	Endogenous	20.25	1.56
FGF6	NM_020996.1	NM_020996.1:420	Endogenous	30.11	2.34
FGF7	NM_002009.3	NM_002009.3:190	Endogenous	32.27	3.52
FGF8	NM_033163.3	NM_033163.3:544	Endogenous	68.99	−3.84
FGF9	NM_002010.2	NM_002010.2:1565	Endogenous	121.14	4.49
FGFR1	NM_015850.2	NM_015850.2:1335	Endogenous	61.58	−5.12
FGFR2	NM_000141.4	NM_000141.4:647	Endogenous	69.30	2.59
FGFR3	NM_022965.2	NM_022965.2:3170	Endogenous	14.81	−1.39
FGFR4	NM_002011.3	NM_002011.3:1585	Endogenous	46.13	2.07
FIGF	NM_004469.2	NM_004469.2:580	Endogenous	70.82	−5.97
FLNA	NM_001456.3	NM_001456.3:7335	Endogenous	32.15	1.64
FLNC	NM_001127487.1	NM_001127487.1:7144	Endogenous	57.05	−1.28
FLT1	NM_002019.4	NM_002019.4:530	Endogenous	4.13	1.17
FLT3	NM_004119.1	NM_004119.1:580	Endogenous	17.60	−1.28
FN1	NM_212482.1	NM_212482.1:1776	Endogenous	87.11	−3.65
FOS	NM_005252.2	NM_005252.2:1475	Endogenous	19.38	−1.46
FOSL1	NM_005438.2	NM_005438.2:280	Endogenous	24.52	−1.34
FOXL2	NM_023067.2	NM_023067.2:1290	Endogenous	15.56	−1.28
FOXO4	NM_005938.2	NM_005938.2:35	Endogenous	60.38	−1.71
FST	NM_006350.2	NM_006350.2:575	Endogenous	112.06	2.34
FTSJ2	NM_013393.1	NM_013393.1:1435	Housekeeping	7.71	1.07
FUBP1	NM_003902.3	NM_003902.3:820	Endogenous	15.70	1.02
FUT8	NM_004480.4	NM_004480.4:2841	Endogenous	27.56	1.61
FZD10	NM_007197.2	NM_007197.2:1810	Endogenous	35.71	−1.71
FZD2	NM_001466.2	NM_001466.2:845	Endogenous	17.36	1.09
FZD3	NM_017412.2	NM_017412.2:435	Endogenous	15.20	1.37
FZD7	NM_003507.1	NM_003507.1:1890	Endogenous	13.18	−1.59
FZD8	NM_031866.1	NM_031866.1:890	Endogenous	54.97	1.17
FZD9	NM_003508.2	NM_003508.2:1320	Endogenous	38.46	−3.84
GADD45A	NM_001924.2	NM_001924.2:865	Endogenous	14.40	1.28
GADD45B	NM_015675.2	NM_015675.2:365	Endogenous	5.82	−1.03
GADD45G	NM_006705.3	NM_006705.3:250	Endogenous	74.05	−12.80
GAS1	NM_002048.2	NM_002048.2:1525	Endogenous	74.60	−5.12
GATA1	NM_002049.2	NM_002049.2:1001	Endogenous	10.12	−1.28
GATA2	NM_032638.3	NM_032638.3:1495	Endogenous	54.87	−3.80
GATA3	NM_001002295.1	NM_001002295.1:2835	Endogenous	12.77	1.37

GDF6	NM_001001557.2	NM_001001557.2:1908	Endogenous	20.51	2.34
GHR	NM_000163.2	NM_000163.2:1835	Endogenous	42.41	−1.28
GLI1	NM_005269.1	NM_005269.1:2885	Endogenous	57.04	1.30
GLI3	NM_000168.5	NM_000168.5:4769	Endogenous	59.75	−5.12
GNA11	NM_002067.1	NM_002067.1:555	Endogenous	23.37	1.44
GNAQ	NM_002072.2	NM_002072.2:1100	Endogenous	10.88	1.20
GNAS	NM_080425.1	NM_080425.1:1910	Endogenous	3.95	1.05
GNG12	NM_018841.3	NM_018841.3:245	Endogenous	11.62	−1.29
GNG4	NM_004485.2	NM_004485.2:215	Endogenous	68.09	−9.15
GNG7	NM_052847.1	NM_052847.1:3920	Endogenous	16.75	−2.13
GNGT1	NM_021955.3	NM_021955.3:260	Endogenous	30.21	−1.99
GPATCH3	NM_022078.2	NM_022078.2:1685	Housekeeping	16.93	1.42
GPC4	NM_001448.2	NM_001448.2:820	Endogenous	116.98	5.58
GRB2	NM_002086.4	NM_002086.4:412	Endogenous	18.79	−1.28
GRIA3	NM_000828.4	NM_000828.4:426	Endogenous	45.29	−1.28
GRIN1	NM_000832.5	NM_000832.5:1290	Endogenous	35.83	1.30
GRIN2A	NM_000833.3	NM_000833.3:4497	Endogenous	49.60	−1.28
GRIN2B	NM_000834.3	NM_000834.3:1144	Endogenous	42.13	1.56
GSK3B	NM_002093.2	NM_002093.2:925	Endogenous	21.71	−1.26
GTF2H3	NM_001516.3	NM_001516.3:70	Endogenous	5.80	1.16
GZMB	NM_004131.3	NM_004131.3:540	Endogenous	53.23	1.95
H2AFX	NM_002105.2	NM_002105.2:1392	Endogenous	11.64	−1.58
H3F3A	NM_002107.3	NM_002107.3:190	Endogenous	19.14	1.18
H3F3C	NM_001013699.2	NM_001013699.2:829	Endogenous	11.46	−1.23
HDAC1	NM_004964.2	NM_004964.2:785	Endogenous	4.64	1.02
HDAC10	NM_032019.5	NM_032019.5:932	Endogenous	11.99	−1.04
HDAC11	NM_024827.3	NM_024827.3:2601	Endogenous	22.50	−1.56
HDAC2	NM_001527.1	NM_001527.1:930	Endogenous	17.57	1.21
HDAC3	NM_003883.2	NM_003883.2:1455	Housekeeping	4.62	1.01
HDAC4	NM_006037.3	NM_006037.3:6965	Endogenous	5.79	−1.25
HDAC5	NM_005474.4	NM_005474.4:3160	Endogenous	32.10	−1.87
HDAC6	NM_006044.2	NM_006044.2:536	Endogenous	30.16	−1.81
HELLS	NM_018063.3	NM_018063.3:2040	Endogenous	19.02	−1.17
HES1	NM_005524.2	NM_005524.2:860	Endogenous	22.39	1.11
HES5	NM_001010926.3	NM_001010926.3:1160	Endogenous	27.68	−2.13
HGF	NM_000601.4	NM_000601.4:550	Endogenous	15.78	1.56
HHEX	NM_002729.4	NM_002729.4:1479	Endogenous	9.82	−1.08
HHIP	NM_022475.1	NM_022475.1:2135	Endogenous	52.19	−1.28
HIST1H3B	NM_003537.3	NM_003537.3:335	Endogenous	16.16	−1.42
HIST1H3G	NM_003534.2	NM_003534.2:348	Endogenous	22.52	−1.58

HIST1H3H	NM_003536.2	NM_003536.2:355	Endogenous	11.11	−1.19
HMGA1	NM_145904.1	NM_145904.1:871	Endogenous	11.08	−1.02
HMGA2	NM_003484.1	NM_003484.1:328	Endogenous	28.48	1.60
HNF1A	NM_000545.4	NM_000545.4:2125	Endogenous	36.77	1.17
HOXA10	NM_018951.3	NM_018951.3:1503	Endogenous	57.78	1.14
HOXA11	NM_005523.5	NM_005523.5:1525	Endogenous	55.84	−3.20
HOXA9	NM_152739.3	NM_152739.3:1015	Endogenous	31.55	3.91
HPGD	NM_001145816.2	NM_001145816.2:96	Endogenous	81.72	1.25
HRAS	NM_005343.2	NM_005343.2:396	Endogenous	27.93	1.46
HSP90B1	NM_003299.1	NM_003299.1:160	Endogenous	29.24	1.41
HSPA1A	NM_005345.5	NM_005345.5:98	Endogenous	45.90	1.27
HSPA2	NM_021979.3	NM_021979.3:2095	Endogenous	28.28	−2.22
HSPA6	NM_002155.3	NM_002155.3:2018	Endogenous	18.07	1.37
HSPB1	NM_001540.3	NM_001540.3:374	Endogenous	1.54	−1.04
IBSP	NM_004967.3	NM_004967.3:876	Endogenous	12.53	1.56
ID1	NM_002165.2	NM_002165.2:345	Endogenous	64.74	1.78
ID2	NM_002166.4	NM_002166.4:505	Endogenous	45.12	1.55
ID4	NM_001546.2	NM_001546.2:2048	Endogenous	47.53	2.73
IDH1	NM_005896.2	NM_005896.2:105	Endogenous	11.32	−1.47
IDH2	NM_002168.2	NM_002168.2:944	Endogenous	25.69	−1.96
IFNA17	NM_021268.2	NM_021268.2:291	Endogenous	37.36	−1.14
IFNA2	NM_000605.3	NM_000605.3:11	Endogenous	21.43	1.17
IFNA7	NM_021057.2	NM_021057.2:0	Endogenous	29.47	1.56
IFNG	NM_000619.2	NM_000619.2:970	Endogenous	53.23	1.95
IGF1	NM_000618.3	NM_000618.3:491	Endogenous	39.24	2.08
IGF1R	NM_000875.2	NM_000875.2:455	Endogenous	48.77	−2.96
IGFBP3	NM_000598.4	NM_000598.4:1255	Endogenous	94.96	−15.02
IKBKB	NM_001556.1	NM_001556.1:1995	Endogenous	21.44	−1.95
IKBKG	NM_003639.2	NM_003639.2:470	Endogenous	10.46	−1.01
IL10	NM_000572.2	NM_000572.2:230	Endogenous	23.76	2.34
IL11	NM_000641.2	NM_000641.2:1145	Endogenous	64.37	−1.46
IL11RA	NM_147162.1	NM_147162.1:400	Endogenous	62.22	−6.40
IL12A	NM_000882.2	NM_000882.2:775	Endogenous	23.99	−1.75
IL12B	NM_002187.2	NM_002187.2:1435	Endogenous	34.06	−1.28
IL12RB2	NM_001559.2	NM_001559.2:1315	Endogenous	44.96	2.34
IL13	NM_002188.2	NM_002188.2:516	Endogenous	112.17	3.91
IL13RA2	NM_000640.2	NM_000640.2:400	Endogenous	43.59	1.95
IL15	NM_172174.1	NM_172174.1:1685	Endogenous	47.83	−1.28
IL19	NM_013371.3	NM_013371.3:1030	Endogenous	73.58	1.95
IL1A	NM_000575.3	NM_000575.3:1085	Endogenous	83.45	−3.58

IL1B	NM_000576.2	NM_000576.2:840	Endogenous	49.27	−4.48
IL1R1	NM_000877.2	NM_000877.2:4295	Endogenous	48.98	−2.43
IL1R2	NM_173343.1	NM_173343.1:113	Endogenous	50.58	−1.83
IL1RAP	NM_002182.2	NM_002182.2:460	Endogenous	18.68	1.45
IL20RA	NM_014432.2	NM_014432.2:535	Endogenous	102.53	3.36
IL20RB	NM_144717.2	NM_144717.2:1575	Endogenous	27.79	−2.06
IL22RA1	NM_021258.2	NM_021258.2:2524	Endogenous	66.51	2.83
IL22RA2	NM_181309.1	NM_181309.1:290	Endogenous	38.01	3.13
IL23A	NM_016584.2	NM_016584.2:411	Endogenous	50.15	−2.05
IL23R	NM_144701.2	NM_144701.2:710	Endogenous	38.35	−1.28
IL24	NM_181339.1	NM_181339.1:1016	Endogenous	28.56	−1.54
IL2RA	NM_000417.1	NM_000417.1:1000	Endogenous	55.29	−2.56
IL2RB	NM_000878.2	NM_000878.2:1980	Endogenous	55.45	−4.27
IL3	NM_000588.3	NM_000588.3:130	Endogenous	27.27	1.07
IL3RA	NM_002183.2	NM_002183.2:745	Endogenous	73.76	−3.84
IL5RA	NM_000564.3	NM_000564.3:210	Endogenous	18.27	1.30
IL6	NM_000600.1	NM_000600.1:220	Endogenous	47.54	1.56
IL6R	NM_000565.2	NM_000565.2:993	Endogenous	18.56	−1.20
IL7	NM_000880.2	NM_000880.2:38	Endogenous	16.56	−1.28
IL7R	NM_002185.2	NM_002185.2:1610	Endogenous	41.43	−1.28
IL8	NM_000584.2	NM_000584.2:25	Endogenous	61.63	−2.90
INHBA	NM_002192.2	NM_002192.2:490	Endogenous	33.39	1.17
INHBB	NM_002193.2	NM_002193.2:1969	Endogenous	2.78	−1.54
IRAK2	NM_001570.3	NM_001570.3:1285	Endogenous	44.16	−1.96
IRAK3	NM_007199.1	NM_007199.1:1735	Endogenous	4.13	−1.28
IRS1	NM_005544.2	NM_005544.2:6224	Endogenous	75.37	3.21
ITGA2	NM_002203.2	NM_002203.2:475	Endogenous	32.29	1.08
ITGA3	NM_005501.2	NM_005501.2:1138	Endogenous	9.73	−1.42
ITGA6	NM_000210.1	NM_000210.1:3065	Endogenous	17.15	−1.27
ITGA7	NM_002206.1	NM_002206.1:1170	Endogenous	38.01	2.08
ITGA8	NM_003638.1	NM_003638.1:1215	Endogenous	48.55	1.17
ITGA9	NM_002207.2	NM_002207.2:2380	Endogenous	43.16	1.56
ITGB3	NM_000212.2	NM_000212.2:4485	Endogenous	42.13	1.17
ITGB4	NM_001005731.1	NM_001005731.1:4151	Endogenous	46.44	−3.19
ITGB6	NM_001282353.1	NM_001282353.1:4124	Endogenous	119.82	5.02
ITGB7	NM_000889.1	NM_000889.1:1278	Endogenous	21.43	−2.99
ITGB8	NM_002214.2	NM_002214.2:2609	Endogenous	39.33	−1.59
JAG1	NM_000214.2	NM_000214.2:915	Endogenous	118.74	4.50
JAG2	NM_145159.1	NM_145159.1:4225	Endogenous	51.73	−2.94
JAK1	NM_002227.1	NM_002227.1:285	Endogenous	39.98	−1.88

JAK2	NM_004972.2	NM_004972.2:455	Endogenous	38.80	1.76
JAK3	NM_000215.2	NM_000215.2:1715	Endogenous	47.97	−3.41
JUN	NM_002228.3	NM_002228.3:140	Endogenous	88.98	3.13
KAT2B	NM_003884.3	NM_003884.3:1220	Endogenous	6.18	1.08
KDM5C	NM_004187.2	NM_004187.2:1170	Endogenous	18.32	1.48
KDM6A	NM_021140.2	NM_021140.2:2590	Endogenous	57.03	2.64
KIT	NM_000222.1	NM_000222.1:5	Endogenous	26.47	−3.20
KITLG	NM_003994.4	NM_003994.4:1155	Endogenous	113.71	7.49
KLF4	NM_004235.4	NM_004235.4:1980	Endogenous	47.59	−1.81
KMT2C	NM_170606.2	NM_170606.2:12505	Endogenous	9.27	−1.05
KMT2D	NM_003482.3	NM_003482.3:6070	Endogenous	36.84	1.64
KRAS	NM_004985.3	NM_004985.3:1790	Endogenous	8.21	1.60
LAMA1	NM_005559.2	NM_005559.2:5230	Endogenous	58.94	−4.08
LAMA3	NM_000227.3	NM_000227.3:4260	Endogenous	145.21	9.92
LAMA5	NM_005560.3	NM_005560.3:787	Endogenous	48.88	2.00
LAMB3	NM_000228.2	NM_000228.2:695	Endogenous	46.36	1.96
LAMB4	NM_007356.2	NM_007356.2:2852	Endogenous	39.31	−1.92
LAMC2	NM_005562.2	NM_005562.2:2819	Endogenous	76.32	−1.97
LAMC3	NM_006059.3	NM_006059.3:2090	Endogenous	28.42	−1.02
LAT	NM_001014987.1	NM_001014987.1:1290	Endogenous	73.67	2.19
LEF1	NM_016269.3	NM_016269.3:1165	Endogenous	60.63	−4.94
LEFTY1	NM_020997.2	NM_020997.2:1405	Endogenous	35.94	−2.30
LEFTY2	NM_003240.2	NM_003240.2:1283	Endogenous	17.28	1.00
LEP	NM_000230.2	NM_000230.2:1875	Endogenous	49.52	−1.28
LEPR	NM_001003679.1	NM_001003679.1:2000	Endogenous	14.79	−1.50
LFNG	NM_001040168.1	NM_001040168.1:717	Endogenous	46.24	1.90
LIF	NM_002309.3	NM_002309.3:1240	Endogenous	23.76	1.48
LIFR	NM_002310.3	NM_002310.3:2995	Endogenous	146.58	23.01
LIG4	NM_002312.3	NM_002312.3:2623	Endogenous	10.42	1.39
LRP2	NM_004525.2	NM_004525.2:12505	Endogenous	13.63	−1.28
LTBP1	NM_000627.3	NM_000627.3:4124	Endogenous	40.27	−2.83
MAD2L2	NM_001127325.1	NM_001127325.1:290	Endogenous	12.67	−1.27
MAML2	NM_032427.1	NM_032427.1:4125	Endogenous	43.73	−2.69
MAP2K1	NM_002755.2	NM_002755.2:970	Endogenous	26.68	−1.77
MAP2K2	NM_030662.2	NM_030662.2:1325	Endogenous	16.08	1.15
MAP2K4	NM_003010.2	NM_003010.2:2830	Endogenous	6.30	1.15
MAP2K6	NM_002758.3	NM_002758.3:555	Endogenous	30.26	−2.26
MAP3K1	NM_005921.1	NM_005921.1:2525	Endogenous	38.06	2.20
MAP3K12	NM_006301.2	NM_006301.2:800	Endogenous	37.08	−2.30
MAP3K13	NM_004721.3	NM_004721.3:965	Endogenous	5.93	−1.14

MAP3K14	NM_003954.1	NM_003954.1:620	Endogenous	14.65	1.33
MAP3K5	NM_005923.3	NM_005923.3:2415	Endogenous	15.04	−1.12
MAP3K8	NM_005204.2	NM_005204.2:2050	Endogenous	27.03	−1.81
MAPK1	NM_138957.2	NM_138957.2:430	Endogenous	11.07	−1.36
MAPK10	NM_002753.2	NM_002753.2:2080	Endogenous	20.19	1.14
MAPK12	NM_002969.3	NM_002969.3:425	Endogenous	70.67	−8.06
MAPK3	NM_001040056.1	NM_001040056.1:580	Endogenous	33.61	−1.82
MAPK8	NM_002750.2	NM_002750.2:945	Endogenous	5.80	1.48
MAPK8IP1	NM_005456.2	NM_005456.2:259	Endogenous	24.19	−2.13
MAPK8IP2	NM_012324.2	NM_012324.2:1885	Endogenous	71.77	−4.98
MAPK9	NM_139068.2	NM_139068.2:365	Endogenous	4.62	−1.07
MAPT	NM_016834.3	NM_016834.3:1205	Endogenous	60.13	−2.56
MCM2	NM_004526.2	NM_004526.2:2945	Endogenous	15.56	−1.57
MCM4	NM_182746.1	NM_182746.1:1200	Endogenous	15.00	−1.64
MCM5	NM_006739.3	NM_006739.3:1580	Endogenous	11.86	−1.57
MCM7	NM_182776.1	NM_182776.1:1325	Endogenous	10.10	−1.11
MDC1	NM_014641.2	NM_014641.2:6719	Endogenous	11.93	−1.19
MDM2	NM_006878.2	NM_006878.2:280	Endogenous	20.60	1.50
MECOM	NM_005241.2	NM_005241.2:3355	Endogenous	102.62	5.27
MED12	NM_005120.2	NM_005120.2:375	Endogenous	8.21	1.04
MEN1	NM_130802.2	NM_130802.2:2480	Endogenous	18.82	−1.38
MET	NM_000245.2	NM_000245.2:405	Endogenous	16.17	−1.39
MFNG	NM_002405.2	NM_002405.2:1681	Endogenous	16.46	1.04
MGMT	NM_002412.3	NM_002412.3:323	Endogenous	35.37	1.57
MLF1	NM_022443.3	NM_022443.3:720	Endogenous	46.94	−1.28
MLH1	NM_000249.2	NM_000249.2:1605	Endogenous	23.43	1.29
MLLT3	NM_004529.2	NM_004529.2:1480	Endogenous	81.32	5.14
MLLT4	NM_005936.2	NM_005936.2:1560	Endogenous	38.37	−2.15
MMP3	NM_002422.3	NM_002422.3:25	Endogenous	60.13	−1.28
MMP7	NM_002423.3	NM_002423.3:311	Endogenous	48.03	1.12
MMP9	NM_004994.2	NM_004994.2:1530	Endogenous	49.58	1.88
MNAT1	NM_002431.2	NM_002431.2:975	Endogenous	26.15	−1.47
MPL	NM_005373.2	NM_005373.2:895	Endogenous	38.18	3.91
MPO	NM_000250.1	NM_000250.1:545	Endogenous	12.46	1.12
MRPS5	NM_031902.3	NM_031902.3:390	Housekeeping	21.80	−1.35
MSH2	NM_000251.1	NM_000251.1:2105	Endogenous	8.98	−1.04
MSH6	NM_000179.1	NM_000179.1:3525	Endogenous	11.90	−1.66
MTMR14	NM_022485.3	NM_022485.3:720	Housekeeping	8.90	1.02
MTOR	NM_004958.2	NM_004958.2:5095	Endogenous	7.05	1.02
MUTYH	NM_012222.2	NM_012222.2:412	Endogenous	44.41	−1.12

MYB	NM_005375.2	NM_005375.2:3145	Endogenous	30.60	1.03
MYC	NM_002467.3	NM_002467.3:1610	Endogenous	21.74	1.22
MYCN	NM_005378.4	NM_005378.4:1545	Endogenous	46.91	−2.53
MYD88	NM_002468.3	NM_002468.3:2145	Endogenous	44.00	1.71
NASP	NM_172164.1	NM_172164.1:2970	Endogenous	15.05	−1.15
NBN	NM_001024688.1	NM_001024688.1:1105	Endogenous	17.01	−1.47
NCOR1	NM_006311.3	NM_006311.3:1390	Endogenous	4.21	−1.09
NF1	NM_000267.2	NM_000267.2:1035	Endogenous	21.58	−1.44
NF2	NM_181828.2	NM_181828.2:455	Endogenous	6.04	1.08
NFATC1	NM_172389.1	NM_172389.1:1984	Endogenous	74.26	−3.84
NFE2L2	NM_006164.3	NM_006164.3:995	Endogenous	3.86	−1.07
NFKB1	NM_003998.2	NM_003998.2:1675	Endogenous	28.69	1.33
NFKBIA	NM_020529.1	NM_020529.1:945	Endogenous	43.15	−1.28
NFKBIZ	NM_001005474.1	NM_001005474.1:2030	Endogenous	59.73	1.92
NGF	NM_002506.2	NM_002506.2:100	Endogenous	40.28	−1.02
NGFR	NM_002507.1	NM_002507.1:2705	Endogenous	66.59	−12.48
NKD1	NM_033119.3	NM_033119.3:2325	Endogenous	53.66	1.79
NODAL	NM_018055.3	NM_018055.3:320	Endogenous	125.93	2.86
NOG	NM_005450.4	NM_005450.4:1543	Endogenous	41.00	−2.21
NOL7	NM_016167.3	NM_016167.3:335	Housekeeping	3.97	1.12
NOS3	NM_000603.4	NM_000603.4:1456	Endogenous	49.19	1.22
NOTCH1	NM_017617.3	NM_017617.3:735	Endogenous	12.84	1.25
NOTCH2	NM_024408.3	NM_024408.3:2842	Endogenous	12.79	1.46
NOTCH3	NM_000435.2	NM_000435.2:1965	Endogenous	32.27	−2.17
NPM1	NM_002520.5	NM_002520.5:10	Endogenous	20.06	−1.53
NPM2	NM_182795.1	NM_182795.1:745	Endogenous	72.34	−1.28
NR4A1	NM_173157.1	NM_173157.1:1575	Endogenous	31.69	−1.42
NR4A3	NM_173198.1	NM_173198.1:2590	Endogenous	34.78	1.56
NRAS	NM_002524.3	NM_002524.3:877	Endogenous	23.22	−1.57
NSD1	NM_022455.4	NM_022455.4:10655	Endogenous	14.59	1.43
NTF3	NM_002527.4	NM_002527.4:151	Endogenous	55.29	−1.28
NTHL1	NM_002528.5	NM_002528.5:476	Endogenous	4.70	1.03
NTRK1	NM_001012331.1	NM_001012331.1:1365	Endogenous	27.68	2.34
NTRK2	NM_001007097.1	NM_001007097.1:1605	Endogenous	32.58	1.56
NUBP1	NM_001278506.1	NM_001278506.1:304	Housekeeping	26.00	−2.00
NUMBL	NM_004756.3	NM_004756.3:591	Endogenous	17.05	1.31
NUPR1	NM_001042483.1	NM_001042483.1:829	Endogenous	77.65	−7.28
OSM	NM_020530.3	NM_020530.3:1662	Endogenous	46.95	1.25
PAK3	NM_002578.2	NM_002578.2:1830	Endogenous	77.09	4.69
PAK7	NM_177990.1	NM_177990.1:615	Endogenous	35.32	1.56

PAX3	NM_013942.3	NM_013942.3:705	Endogenous	89.21	−1.28
PAX5	NM_016734.1	NM_016734.1:2288	Endogenous	40.63	1.30
PAX8	NM_013953.3	NM_013953.3:3465	Endogenous	65.85	−2.56
PBRM1	NM_181042.3	NM_181042.3:6100	Endogenous	46.04	2.27
PBX1	NM_002585.2	NM_002585.2:368	Endogenous	9.57	−1.25
PBX3	NM_006195.5	NM_006195.5:2050	Endogenous	28.89	−2.37
PCK1	NM_002591.2	NM_002591.2:1870	Endogenous	25.94	−1.28
PCNA	NM_002592.2	NM_002592.2:280	Endogenous	21.27	1.02
PDGFA	NM_002607.5	NM_002607.5:2460	Endogenous	16.05	−1.08
PDGFB	NM_033016.2	NM_033016.2:1480	Endogenous	18.02	1.22
PDGFC	NM_016205.1	NM_016205.1:10	Endogenous	66.54	−5.44
PDGFD	NM_025208.4	NM_025208.4:1120	Endogenous	30.83	3.91
PDGFRA	NM_006206.3	NM_006206.3:1925	Endogenous	151.49	14.84
PDGFRB	NM_002609.3	NM_002609.3:840	Endogenous	36.47	−1.28
PGF	NM_002632.5	NM_002632.5:1047	Endogenous	20.91	−1.02
PHF6	NM_032335.3	NM_032335.3:85	Endogenous	9.86	−1.21
PIAS1	NM_016166.1	NM_016166.1:1870	Housekeeping	6.97	−1.11
PIK3CA	NM_006218.2	NM_006218.2:2445	Endogenous	42.94	−2.17
PIK3CB	NM_006219.1	NM_006219.1:2945	Endogenous	18.90	−1.39
PIK3CD	NM_005026.3	NM_005026.3:95	Endogenous	39.31	−1.76
PIK3CG	NM_002649.2	NM_002649.2:2125	Endogenous	29.62	−1.28
PIK3R1	NM_181504.2	NM_181504.2:1105	Endogenous	110.52	4.67
PIK3R2	NM_005027.2	NM_005027.2:3100	Endogenous	26.15	−1.26
PIK3R3	NM_003629.3	NM_003629.3:1800	Endogenous	15.63	−1.11
PIK3R4	NM_014602.1	NM_014602.1:3620	Housekeeping	9.54	−1.14
PIK3R5	NM_001142633.1	NM_001142633.1:3335	Endogenous	54.15	−4.16
PIM1	NM_002648.2	NM_002648.2:1630	Endogenous	34.70	−1.76
PITX2	NM_000325.5	NM_000325.5:1381	Endogenous	57.14	1.56
PKMYT1	NM_004203.3	NM_004203.3:780	Endogenous	1.39	1.12
PLA1A	NM_015900.2	NM_015900.2:1250	Endogenous	55.84	−1.92
PLA2G10	NM_003561.1	NM_003561.1:779	Endogenous	16.84	3.13
PLA2G2A	NM_000300.2	NM_000300.2:715	Endogenous	95.45	3.15
PLA2G3	NM_015715.3	NM_015715.3:2415	Endogenous	10.89	−1.14
PLA2G4A	NM_024420.2	NM_024420.2:1763	Endogenous	16.68	−1.54
PLA2G4C	NM_003706.2	NM_003706.2:2310	Endogenous	87.50	3.13
PLA2G4E	NM_001206670.1	NM_001206670.1:4174	Endogenous	9.59	3.13
PLA2G4F	NM_213600.2	NM_213600.2:3140	Endogenous	85.21	−5.76
PLA2G5	NM_000929.2	NM_000929.2:1375	Endogenous	45.92	−2.56
PLAT	NM_000931.2	NM_000931.2:1334	Endogenous	46.23	−3.47
PLAU	NM_002658.2	NM_002658.2:793	Endogenous	47.62	−2.13

PLCB1	NM_182734.1	NM_182734.1:170	Endogenous	21.04	−1.69
PLCB4	NM_000933.3	NM_000933.3:215	Endogenous	119.41	5.05
PLCE1	NM_001165979.1	NM_001165979.1:392	Endogenous	135.91	6.41
PLCG2	NM_002661.2	NM_002661.2:525	Endogenous	49.96	−2.45
PLD1	NM_002662.3	NM_002662.3:1265	Endogenous	59.40	−7.25
PML	NM_002675.3	NM_002675.3:281	Endogenous	15.16	−1.02
POLB	NM_002690.1	NM_002690.1:145	Endogenous	31.33	−1.90
POLD1	NM_002691.2	NM_002691.2:2392	Endogenous	20.75	1.29
POLD4	NM_021173.2	NM_021173.2:470	Endogenous	10.67	1.05
POLE2	NM_002692.2	NM_002692.2:1420	Endogenous	11.94	−1.04
POLR2D	NM_004805.3	NM_004805.3:1476	Endogenous	4.46	−1.06
POLR2H	NM_001278698.1	NM_001278698.1:940	Endogenous	18.36	−1.42
POLR2J	NM_006234.4	NM_006234.4:618	Endogenous	28.75	1.51
PPARG	NM_015869.3	NM_015869.3:1035	Endogenous	20.62	−1.74
PPARGC1A	NM_013261.3	NM_013261.3:1505	Endogenous	70.74	1.49
PPP2CB	NM_001009552.1	NM_001009552.1:1075	Endogenous	4.79	1.76
PPP2R1A	NM_014225.3	NM_014225.3:1440	Endogenous	19.12	1.25
PPP2R2B	NM_181676.2	NM_181676.2:690	Endogenous	35.32	1.56
PPP2R2C	NM_181876.2	NM_181876.2:2215	Endogenous	6.72	−1.60
PPP3CA	NM_000944.4	NM_000944.4:3920	Endogenous	25.21	−1.70
PPP3CB	NM_001142354.1	NM_001142354.1:1690	Endogenous	25.13	−1.05
PPP3CC	NM_005605.3	NM_005605.3:1460	Endogenous	13.83	1.10
PPP3R1	NM_000945.3	NM_000945.3:2385	Endogenous	9.74	−1.10
PPP3R2	NM_147180.2	NM_147180.2:1165	Endogenous	52.57	−3.84
PRDM1	NM_182907.1	NM_182907.1:310	Endogenous	28.14	−1.03
PRKAA2	NM_006252.2	NM_006252.2:975	Endogenous	9.30	3.91
PRKACA	NM_002730.3	NM_002730.3:400	Endogenous	11.92	−1.24
PRKACB	NM_182948.2	NM_182948.2:805	Endogenous	117.09	3.52
PRKACG	NM_002732.2	NM_002732.2:775	Endogenous	53.15	3.13
PRKAR1B	NM_001164759.1	NM_001164759.1:1112	Endogenous	20.95	1.31
PRKAR2A	NM_004157.2	NM_004157.2:1590	Endogenous	142.53	13.33
PRKAR2B	NM_002736.2	NM_002736.2:1350	Endogenous	19.83	−1.71
PRKCA	NM_002737.2	NM_002737.2:5560	Endogenous	28.68	2.41
PRKCB	NM_212535.1	NM_212535.1:1750	Endogenous	58.00	1.56
PRKCG	NM_002739.3	NM_002739.3:445	Endogenous	12.07	1.95
PRKDC	NM_006904.6	NM_006904.6:12750	Endogenous	16.99	−1.40
PRKX	NM_005044.1	NM_005044.1:2590	Endogenous	40.38	1.30
PRL	NM_000948.3	NM_000948.3:835	Endogenous	30.83	1.95
PRLR	NM_001204318.1	NM_001204318.1:563	Endogenous	52.42	1.12
PRMT8	NM_019854.3	NM_019854.3:1900	Endogenous	44.96	2.34

PROM1	NM_006017.1	NM_006017.1:925	Endogenous	63.88	−1.28
PRPF38A	NM_032864.3	NM_032864.3:335	Housekeeping	3.12	−1.01
PTCH1	NM_000264.3	NM_000264.3:5420	Endogenous	5.09	−1.11
PTCRA	NM_138296.2	NM_138296.2:507	Endogenous	23.28	−2.90
PTEN	NM_000314.3	NM_000314.3:1675	Endogenous	7.48	1.04
PTPN11	NM_002834.3	NM_002834.3:1480	Endogenous	5.40	−1.11
PTPN5	NM_001039970.1	NM_001039970.1:2354	Endogenous	69.87	3.91
PTPRR	NM_001207015.1	NM_001207015.1:1652	Endogenous	49.43	6.25
PTTG2	NM_006607.2	NM_006607.2:5	Endogenous	30.35	−2.20
RAC1	NM_198829.1	NM_198829.1:1250	Endogenous	25.50	−1.68
RAC2	NM_002872.3	NM_002872.3:1069	Endogenous	42.89	−2.42
RAC3	NM_005052.2	NM_005052.2:702	Endogenous	34.95	1.48
RAD21	NM_006265.2	NM_006265.2:1080	Endogenous	34.55	−2.02
RAD50	NM_005732.2	NM_005732.2:5397	Endogenous	13.96	1.10
RAD51	NM_133487.2	NM_133487.2:566	Endogenous	25.74	−1.80
RAD52	NM_134424.2	NM_134424.2:297	Endogenous	64.86	2.70
RAF1	NM_002880.2	NM_002880.2:1990	Endogenous	5.05	−1.13
RASA4	NM_001079877.2	NM_001079877.2:5330	Endogenous	60.41	−3.75
RASAL1	NM_004658.1	NM_004658.1:2900	Endogenous	81.96	6.88
RASGRF1	NM_153815.2	NM_153815.2:2443	Endogenous	82.79	−4.08
RASGRF2	NM_006909.1	NM_006909.1:2675	Endogenous	16.56	−3.84
RASGRP1	NM_005739.3	NM_005739.3:365	Endogenous	100.48	6.25
RASGRP2	NM_001098670.1	NM_001098670.1:1978	Endogenous	15.84	−2.19
RB1	NM_000321.1	NM_000321.1:2110	Endogenous	12.35	1.11
RBM45	NM_152945.2	NM_152945.2:1080	Housekeeping	21.12	2.14
RBX1	NM_014248.2	NM_014248.2:162	Endogenous	17.08	−1.55
RELA	NM_021975.3	NM_021975.3:1990	Endogenous	30.50	−1.37
RELN	NM_005045.2	NM_005045.2:345	Endogenous	49.52	−1.28
RET	NM_020630.4	NM_020630.4:2911	Endogenous	32.98	1.04
RFC3	NM_002915.3	NM_002915.3:740	Endogenous	36.22	1.41
RFC4	NM_181573.2	NM_181573.2:1035	Endogenous	36.39	−2.15
RHOA	NM_001664.2	NM_001664.2:1230	Endogenous	36.75	1.55
RIN1	NM_004292.2	NM_004292.2:2572	Endogenous	40.17	−2.96
RNF43	NM_017763.4	NM_017763.4:725	Endogenous	26.49	1.32
RPA3	NM_002947.3	NM_002947.3:990	Endogenous	15.56	−1.28
RPS27A	NM_002954.5	NM_002954.5:650	Endogenous	5.74	−1.09
RPS6KA5	NM_004755.2	NM_004755.2:855	Endogenous	42.65	−1.92
RPS6KA6	NM_014496.1	NM_014496.1:2250	Endogenous	51.60	−1.60
RRAS2	NM_001102669.2	NM_001102669.2:1785	Endogenous	80.25	3.43
RUNX1	NM_001754.4	NM_001754.4:635	Endogenous	33.46	2.51

RUNX1T1	NM_004349.2	NM_004349.2:1085	Endogenous	37.89	1.56
RXRG	NM_006917.3	NM_006917.3:1105	Endogenous	71.28	−1.28
SAP130	NM_024545.3	NM_024545.3:3090	Housekeeping	12.66	−1.08
SETBP1	NM_015559.2	NM_015559.2:704	Endogenous	49.52	−1.28
SETD2	NM_014159.6	NM_014159.6:6160	Endogenous	44.88	2.02
SF3A3	NM_006802.2	NM_006802.2:2060	Housekeeping	7.66	−1.14
SF3B1	NM_001005526.1	NM_001005526.1:0	Endogenous	10.22	−1.15
SFN	NM_006142.3	NM_006142.3:579	Endogenous	27.53	−1.52
SFRP1	NM_003012.3	NM_003012.3:3320	Endogenous	63.51	1.09
SFRP2	NM_003013.2	NM_003013.2:840	Endogenous	10.12	−1.28
SFRP4	NM_003014.2	NM_003014.2:1060	Endogenous	42.13	−2.56
SGK2	NM_170693.1	NM_170693.1:1485	Endogenous	42.49	−1.61
SHC1	NM_183001.4	NM_183001.4:3355	Endogenous	21.18	−1.28
SHC2	NM_012435.2	NM_012435.2:698	Endogenous	122.08	8.13
SHC3	NM_016848.5	NM_016848.5:6155	Endogenous	20.19	1.56
SHC4	NM_203349.2	NM_203349.2:1570	Endogenous	12.53	−2.24
SIN3A	NM_015477.1	NM_015477.1:1605	Endogenous	28.59	−1.83
SIRT4	NM_012240.1	NM_012240.1:915	Endogenous	45.80	−2.29
SIX1	NM_005982.3	NM_005982.3:25	Endogenous	47.46	−1.28
SKP1	NM_170679.2	NM_170679.2:630	Endogenous	7.80	−1.20
SKP2	NM_005983.2	NM_005983.2:615	Endogenous	17.08	1.21
SLC4A1AP	NM_018158.2	NM_018158.2:980	Housekeeping	19.54	−1.24
SMAD2	NM_001003652.1	NM_001003652.1:4500	Endogenous	25.46	1.04
SMAD3	NM_005902.3	NM_005902.3:4220	Endogenous	19.33	−1.65
SMAD4	NM_005359.3	NM_005359.3:1370	Endogenous	7.55	1.13
SMAD9	NM_005905.2	NM_005905.2:1595	Endogenous	86.94	−9.60
SMARCA4	NM_003072.3	NM_003072.3:5400	Endogenous	22.01	−1.65
SMARCB1	NM_003073.3	NM_003073.3:1060	Endogenous	15.04	−1.01
SMC1A	NM_006306.2	NM_006306.2:2320	Endogenous	24.24	1.13
SMC1B	NM_148674.3	NM_148674.3:3600	Endogenous	13.80	1.17
SMC3	NM_005445.3	NM_005445.3:3525	Endogenous	23.80	−1.72
SMO	NM_005631.3	NM_005631.3:1615	Endogenous	20.04	−1.76
SOCS1	NM_003745.1	NM_003745.1:1025	Endogenous	67.32	−3.15
SOCS2	NM_003877.3	NM_003877.3:1020	Endogenous	149.70	19.89
SOCS3	NM_003955.3	NM_003955.3:1870	Endogenous	51.48	2.51
SOS1	NM_005633.2	NM_005633.2:1635	Endogenous	15.57	−1.52
SOS2	NM_006939.2	NM_006939.2:3845	Endogenous	11.84	−1.54
SOST	NM_025237.2	NM_025237.2:980	Endogenous	29.47	1.17
SOX17	NM_022454.3	NM_022454.3:1374	Endogenous	18.60	−1.65
SOX9	NM_000346.2	NM_000346.2:2135	Endogenous	11.57	1.12

SP1	NM_003109.1	NM_003109.1:5970	Endogenous	17.20	−1.60
SPOP	NM_001007226.1	NM_001007226.1:2726	Endogenous	15.64	−1.05
SPP1	NM_000582.2	NM_000582.2:760	Endogenous	10.40	−1.46
SPRY1	NM_005841.1	NM_005841.1:810	Endogenous	31.56	−1.47
SPRY2	NM_005842.2	NM_005842.2:85	Endogenous	50.53	1.85
SPRY4	NM_030964.3	NM_030964.3:1900	Endogenous	12.31	−1.49
SRSF2	NM_003016.3	NM_003016.3:312	Endogenous	35.96	1.63
SSX1	NM_005635.2	NM_005635.2:148	Endogenous	27.19	−1.60
STAG2	NM_001042749.1	NM_001042749.1:4040	Endogenous	9.01	−1.03
STAT1	NM_007315.2	NM_007315.2:205	Endogenous	20.95	1.16
STAT3	NM_139276.2	NM_139276.2:4535	Endogenous	37.63	1.85
STAT4	NM_003151.2	NM_003151.2:789	Endogenous	32.72	−1.28
STK11	NM_000455.4	NM_000455.4:2060	Endogenous	28.03	1.33
STMN1	NM_203401.1	NM_203401.1:478	Endogenous	19.22	−1.93
SUV39H2	NM_024670.3	NM_024670.3:2035	Endogenous	2.37	1.08
SYK	NM_003177.3	NM_003177.3:1685	Endogenous	56.66	2.23
TBL1XR1	NM_024665.4	NM_024665.4:915	Endogenous	14.98	−1.38
TCF3	NM_003200.2	NM_003200.2:4325	Endogenous	50.07	1.63
TCF7L1	NM_031283.1	NM_031283.1:2215	Endogenous	28.76	−1.02
TCL1B	NM_004918.2	NM_004918.2:319	Endogenous	29.47	1.17
TET2	NM_001127208.2	NM_001127208.2:2882	Endogenous	13.97	1.08
TFDP1	NM_007111.4	NM_007111.4:1826	Endogenous	45.33	1.58
TGFB1	NM_000660.3	NM_000660.3:1260	Endogenous	19.47	1.44
TGFB2	NM_003238.2	NM_003238.2:1125	Endogenous	23.40	−1.79
TGFB3	NM_003239.2	NM_003239.2:2825	Endogenous	19.16	1.17
TGFBR2	NM_001024847.1	NM_001024847.1:1760	Endogenous	13.57	−1.29
THBS1	NM_003246.2	NM_003246.2:3465	Endogenous	96.32	7.06
THBS4	NM_003248.3	NM_003248.3:985	Endogenous	57.84	−3.84
THEM4	NM_053055.4	NM_053055.4:764	Endogenous	59.76	2.34
TIAM1	NM_003253.2	NM_003253.2:5620	Endogenous	34.70	−1.85
TLK2	NM_006852.2	NM_006852.2:2335	Housekeeping	5.77	1.08
TLR2	NM_003264.3	NM_003264.3:180	Endogenous	61.64	−1.02
TLR4	NM_138554.2	NM_138554.2:2570	Endogenous	141.52	12.76
TLX1	NM_005521.3	NM_005521.3:1915	Endogenous	48.22	−1.60
TMPRSS2	NM_005656.2	NM_005656.2:1290	Endogenous	131.43	6.29
TMUB2	NM_024107.2	NM_024107.2:1485	Housekeeping	13.07	−1.23
TNC	NM_002160.3	NM_002160.3:4	Endogenous	22.28	1.38
TNF	NM_000594.2	NM_000594.2:1010	Endogenous	112.83	−3.84
TNFAIP3	NM_006290.2	NM_006290.2:260	Endogenous	65.20	−1.38
TNFRSF10A	NM_003844.2	NM_003844.2:950	Endogenous	24.19	1.64

TNFRSF10B	NM_003842.3	NM_003842.3:565	Endogenous	22.46	−1.09
TNFRSF10C	NM_003841.2	NM_003841.2:5	Endogenous	31.97	−2.38
TNFRSF10D	NM_003840.3	NM_003840.3:2380	Endogenous	27.25	1.21
TNFSF10	NM_003810.2	NM_003810.2:115	Endogenous	69.20	−4.27
TNN	NM_022093.1	NM_022093.1:3580	Endogenous	46.10	3.13
TNR	NM_003285.2	NM_003285.2:3154	Endogenous	12.46	−1.02
TP53	NM_000546.2	NM_000546.2:1330	Endogenous	17.02	−1.87
TPO	NM_175722.1	NM_175722.1:2400	Endogenous	101.14	3.13
TRAF7	NM_032271.2	NM_032271.2:1105	Endogenous	12.92	−1.40
TRIM39	NM_021253.3	NM_021253.3:3140	Housekeeping	20.33	1.26
TSC1	NM_000368.3	NM_000368.3:100	Endogenous	12.09	1.07
TSHR	NM_001018036.2	NM_001018036.2:735	Endogenous	30.84	1.95
TSLP	NM_033035.4	NM_033035.4:899	Endogenous	41.18	1.56
TSPAN7	NM_004615.3	NM_004615.3:725	Endogenous	54.97	2.34
TTC31	NR_027749.1	NR_027749.1:2720	Housekeeping	23.51	−1.54
TTK	NM_003318.3	NM_003318.3:1200	Endogenous	13.80	−1.50
U2AF1	NM_001025203.1	NM_001025203.1:400	Endogenous	7.93	−1.10
UBB	NM_018955.2	NM_018955.2:15	Endogenous	15.43	1.24
UBE2T	NM_014176.3	NM_014176.3:595	Endogenous	4.41	−1.47
USP39	NM_001256725.1	NM_001256725.1:806	Housekeeping	15.25	−1.28
UTY	NM_007125.3	NM_007125.3:450	Endogenous	39.31	−1.92
VEGFA	NM_001025366.1	NM_001025366.1:1325	Endogenous	28.54	−1.48
VEGFC	NM_005429.2	NM_005429.2:565	Endogenous	72.35	−1.28
VHL	NM_000551.2	NM_000551.2:1280	Endogenous	8.40	1.14
VPS33B	NM_018668.3	NM_018668.3:2140	Housekeeping	17.32	−1.61
WEE1	NM_003390.3	NM_003390.3:1225	Endogenous	17.57	−1.79
WHSC1	NM_007331.1	NM_007331.1:1260	Endogenous	16.68	−1.57
WHSC1L1	NM_017778.2	NM_017778.2:485	Endogenous	20.90	−1.50
WIF1	NM_007191.2	NM_007191.2:765	Endogenous	85.05	1.56
WNT10A	NM_025216.2	NM_025216.2:2255	Endogenous	31.41	−1.73
WNT10B	NM_003394.2	NM_003394.2:2070	Endogenous	50.71	2.29
WNT11	NM_004626.2	NM_004626.2:960	Endogenous	63.46	−5.28
WNT16	NM_057168.1	NM_057168.1:1621	Endogenous	19.31	−2.56
WNT2	NM_003391.2	NM_003391.2:2014	Endogenous	21.35	−1.28
WNT2B	NM_024494.1	NM_024494.1:1530	Endogenous	47.62	−3.52
WNT3	NM_030753.3	NM_030753.3:1335	Endogenous	22.77	1.06
WNT4	NM_030761.3	NM_030761.3:625	Endogenous	52.42	−4.48
WNT5A	NM_003392.3	NM_003392.3:475	Endogenous	19.83	2.34
WNT5B	NM_032642.2	NM_032642.2:1745	Endogenous	135.27	10.00
WNT6	NM_006522.3	NM_006522.3:1200	Endogenous	29.04	1.30

WNT7A	NM_004625.3	NM_004625.3:325	Endogenous	18.15	1.41
WNT7B	NM_058238.1	NM_058238.1:1535	Endogenous	53.07	−3.98
WT1	NM_000378.3	NM_000378.3:2160	Endogenous	72.97	4.69
XPA	NM_000380.3	NM_000380.3:265	Endogenous	5.02	−1.09
XRCC4	NM_003401.3	NM_003401.3:772	Endogenous	19.04	1.26
ZAK	NM_016653.2	NM_016653.2:995	Endogenous	9.59	−1.24
ZBTB16	NM_006006.4	NM_006006.4:1585	Endogenous	48.76	−1.28
ZBTB32	NM_014383.1	NM_014383.1:1620	Endogenous	56.97	1.04
ZC3H14	NM_001160103.1	NM_001160103.1:2690	Housekeeping	68.05	2.60
ZIC2	NM_007129.2	NM_007129.2:1849	Endogenous	50.32	−3.84
ZKSCAN5	NM_014569.3	NM_014569.3:3688	Housekeeping	9.88	1.14
ZNF143	NM_003442.5	NM_003442.5:925	Housekeeping	6.61	−1.15
ZNF346	NM_012279.2	NM_012279.2:2260	Housekeeping	21.09	1.78
ZNF384	NM_133476.3	NM_133476.3:300	Housekeeping	8.73	1.46
